# Supplementary material for: Adherence and clinical outcomes for twice-daily versus once-daily dosing of non-vitamin K antagonist oral anticoagulants in patients with atrial fibrillation: Is dosing frequency important?
Source: PLoS One. 2023 Mar 30;18(3):e0283478. doi: 10.1371/journal.pone.0283478 (PMC10062560; doi:10.1371/journal.pone.0283478)
Supplement: S1 Table — (DOCX) [file pone.0283478.s001.docx]

S1 Table. Definitions of atrial fibrillation, comorbidities, medications, and clinical outcomes

|  | **ICD-10-CM code^*^** | **Diagnostic definition** |
| --- | --- | --- |
| **Diagnosis** |  |  |
| Atrial fibrillation | I480, I481, I482, I489 |  |
| Comorbidities |  |  |
| *ESRD* | N18-19 | diagnosis code plus dialysis |
| *liver failure or LC* | K72, K74,  K702-704, K711 |  |
| *malignancy* | C00-C97 |  |
| *hyperthyroidism* | E05, E063 |  |
| *hypertension* | I10-13, I15 | diagnosis code plus anti-hypertensive medications (CCB, ACEI/ARB, beta blocker, thiazide/thiazide like drugs, aldosterone antagonist, alpha blocker) |
| *diabetes mellitus* | E10-14 | diagnosis code plus anti-diabetic medications (metformin, sulfonylurea, meglitinides, thiazolidinedione, dipeptidyl peptidase-4 inhibitor, glucagon-like peptide-1 receptor agonist, alpha- glucosidase inhibitor, sodium-glucose cotransporter-2 inhibitor, insulin) |
| *dyslipidemia* | E78 | 1) diagnosis code plus medications including fibrate, Omega-3, and nicotinic acid  2) statin user |
| *myocardial infarction* | I21-I24, I252, I255, I256 |  |
| *stroke* | I60-66 |  |
| *thromboembolism* | I26, I822, I823, I828, I829 |  |
| *arterial diseases* | I67-I68 |  |
| *heart failure* | I50, I130, I132, I110, I255 | diagnosis code plus medication including ACEI/ARB, beta blocker, and diuretics |
| *CKD* | N18-19 |  |
| **Medications** |  |  |
| *antiplatelet agent* |  | aspirin, clopidogrel, cilostazol, ticlopidine, prasugrel, ticagrelor, ticlopidine, triflusal |
| *statin* |  | atorvastatin, rosuvastatin, simvastatin, fluvastatin, pitavastatin, pravastatin, cerivastatin |
| *ACEI/ARB* |  | alacepril, benazepril, captopril, cilazapril, delapril, enalapril, fosinopril, lisinopril, moexipril, ramipril, imidapril, perindopril, quinapril, spirapril, trandolapril, zofenopril, valsartan, losartan, azilsartan, candesartan, telmisartan, eprosartan, fimasartan, irbesartan, olmesartan |
| *beta blocker* |  | acebutolol, atenolol, betaxolol, bevantolol, bisoprolol, carteolol, carvedilol, celiprolol, cloranolol, metoprolol, nadolol, nebivolol, pindolol, propranolol, tertatolol |
| *CCB* |  | amlodipine, nifedipine, benidipine, cilnidipine, efonidipine, felodipine, gallopamil, isradipine, lacidipine, lercanidipine, manidipine, nimodipin, nisoldipine, nitrendipine |
| *diuretics* |  | chlorthalidone, azosemide, furosemide, hydrochlorothiazide, spironolactone, indapamide, mefruside, metolazone, torasemide indapamide, metolazone, amiloride |
| **Clinical outcomes** |  |  |
| *stroke* | I60-66 | diagnosis code plus admission plus brain MRI/CT |
| *AMI* | I21-I23 | diagnosis code plus admission plus percutaneous coronary intervention or coronary angiography |

^*^Admission or outpatient department ≥2; ICD-10-CM, the International Classification of Disease-10th Revision-Clinical Modification; ESRD, end-stage renal disease; LC, liver cirrhosis; CCB, calcium channel blocker; ACEI/ARB, angiotensin converting enzyme inhibitor or angiotensin receptor blocker; CKD, chronic kidney disease; MRI/CT, magnetic resonance imaging or computed tomography; AMI, acute myocardial infarction
