# Supplementary material for: Adherence and clinical outcomes for twice-daily versus once-daily dosing of non-vitamin K antagonist oral anticoagulants in patients with atrial fibrillation: Is dosing frequency important?
Source: PLoS One. 2023 Mar 30;18(3):e0283478. doi: 10.1371/journal.pone.0283478 (PMC10062560; doi:10.1371/journal.pone.0283478)
Supplement: S2 Table — (DOCX) [file pone.0283478.s002.docx]

S2 Table. Clinical characteristics according to NOAC adherence before and after propensity score matching for composite outcome

| **Characteristics** | **Overall population** | | | | **Propensity score-matched population**^*^ | | |
| --- | --- | --- | --- | --- | --- | --- | --- |
|  | High adherence | Low adherence | p value | *d_before_* | High adherence | Low adherence | *d_after_* |
| Subjects, n | 31,921 | 1,594 |  |  | 7,970 | 1,594 |  |
| Age, years | 72.3 ± 10.7 | 72.6 ± 13.2 | 0.279 | 0.025 | 72.5 ± 11.0 | 72.6 ± 13.2 | 0.007 |
| Male, n(%) | 16,788 (53) | 832 (52) | 0.758 | 0.008 | 4,127 (52) | 832 (52) | 0.008 |
| Medical history, n(%) |  |  |  |  |  |  |  |
| hypertension | 25,654 (80) | 1,229 (77) | 0.002 | 0.08 | 6,112 (77) | 1229 (77) | 0.010 |
| diabetes | 8,181 (26) | 360 (23) | 0.006 | 0.071 | 1,747 (22) | 360 (23) | 0.016 |
| dyslipidemia | 19,172 (60) | 780 (49) | <0.001 | 0.225 | 3,870 (49) | 780 (49) | 0.008 |
| myocardial infarction | 2,785 (9) | 156 (10) | 0.147 | 0.037 | 736 (9) | 156 (10) | 0.019 |
| stroke | 9,966 (31) | 470 (29) | 0.15 | 0.038 | 2,365 (30) | 470 (29) | 0.004 |
| thromboembolism | 1,630 (5) | 118 (7) | <0.001 | 0.095 | 567 (7) | 118 (7) | 0.012 |
| arterial diseases^†^ | 5,730 (18) | 308 (19) | 0.171 | 0.035 | 1,482 (19) | 308 (19) | 0.019 |
| heart failure | 14,592 (46) | 799 (50) | 0.001 | 0.088 | 3,789 (48) | 799 (50) | 0.006 |
| CKD | 1,287 (4) | 90 (6) | 0.002 | 0.075 | 434 (5) | 90 (6) | 0.009 |
| CHA2DS2-VASc | 3.5 ± 1.9 | 3.8 ± 2.0 | <0.001 |  |  |  |  |
| 0-1, n(%) | 4,276 (13) | 197 (12) | 0.250 | 0.031 | 919 (12) | 197 (13) | 0.011 |
| 2-3, n(%) | 12,423 (39) | 556 (35) | 0.001 | 0.084 | 2,838 (36) | 556 (35) | 0.006 |
| ≥4, n(%) | 15,222 (48) | 841(53) | <0.001 | 0.102 | 4,213 (53) | 841 (53) | 0.001 |
| Medications, n(%) |  |  |  |  |  |  |  |
| low dosing NOAC | 15,500(49) | 796 (50) | 0.292 | 0.028 | 3,942 (49) | 796 (50) | 0.010 |
| antiplatelet agent | 12,328 (39) | 589 (37) | 0.190 | 0.034 | 3,026 (38) | 589 (37) | 0.016 |
| statin | 19,388 (61) | 824 (52) | <0.001 | 0.183 | 4,103 (51) | 824 (52) | 0.004 |
| ACEI/ARB | 18,272 (57) | 880 (55) | 0.114 | 0.041 | 4,490 (56) | 880 (55) | 0.023 |
| beta blocker | 15,500 (48) | 791 (50) | 0.411 | 0.021 | 3,910 (49) | 791(50) | 0.011 |
| CCB | 13,506 (42) | 650 (41) | 0.232 | 0.031 | 3,270 (41) | 650 (41) | 0.005 |
| diuretics | 3,218 (10) | 199 (12) | 0.003 | 0.076 | 968 (12) | 199 (12) | 0.011 |

NOAC, non-vitamin K antagonist oral anticoagulant; ^*^matching covariates, including demographics, CHA2DS2-VASc score, and medical information data between patients with high and low adherence; *d_before_*, standardized difference before propensity score matching; *d_after_*, standardized difference after propensity score matching; ^†^stenosis or thrombosis of aortic and peripheral arteries; CKD, chronic kidney disease; ACEI/ARB, angiotensin-converting enzyme inhibitor or angiotensin-receptor blocker; CCB, calcium channel blocker
