# Supplementary material for: Adherence and clinical outcomes for twice-daily versus once-daily dosing of non-vitamin K antagonist oral anticoagulants in patients with atrial fibrillation: Is dosing frequency important?
Source: PLoS One. 2023 Mar 30;18(3):e0283478. doi: 10.1371/journal.pone.0283478 (PMC10062560; doi:10.1371/journal.pone.0283478)
Supplement: S3 Table — (DOCX) [file pone.0283478.s003.docx]

S3 Table. Baseline characteristics and standardized differences after propensity score matching for composite outcome between patients with high (PDC ≥80%) and low (PDC <80%) adherence to each NOAC

| **Characteristics** | **Apixaban** | | | | **Dabigatran** | | | | **Edox/Riva** | | | |
| --- | --- | --- | --- | --- | --- | --- | --- | --- | --- | --- | --- | --- |
|  | PDC ≥80% | PDC <80% | *d_before_* | *d_after_* | PDC ≥80% | PDC <80% | *d_before_* | *d_after_* | PDC ≥80% | PDC <80% | *d_before_* | *d_after_* |
| Subjects, n | 2,030 | 407 |  |  | 2,277 | 456 |  |  | 3,655 | 731 |  |  |
| Age, years | 73 ± 12 | 73 ± 13 | 0.048 | 0.004 | 71 ± 11 | 71 ± 13 | 0.007 | <0.001 | 73 ± 11 | 73 ± 13 | 0.107 | 0.009 |
| Male, n(%) | 1,110 (55) | 215 (53) | 0.092 | 0.037 | 1,194 (52) | 238 (52) | 0.082 | 0.005 | 1,907 (52) | 379 (52) | 0.036 | 0.007 |
| Medical history, n(%) |  |  |  |  |  |  |  |  |  |  |  |  |
| hypertension | 1,601 (79) | 316 (78) | 0.074 | 0.030 | 1,743 (77) | 345 (76) | 0.094 | 0.021 | 2,824 (77) | 568 (77) | 0.070 | 0.011 |
| diabetes | 487 (24) | 96 (24) | 0.064 | 0.009 | 523 (23) | 103 (23) | 0.065 | 0.009 | 809 (22) | 161 (22) | 0.077 | 0.003 |
| dyslipidemia | 986 (49) | 195 (48) | 0.267 | 0.013 | 1,123 (49) | 230 (50) | 0.245 | 0.023 | 1,777 (49) | 355 (49) | 0.197 | 0.001 |
| myocardial infarction | 230 (11) | 48 (12) | 0.040 | 0.015 | 204 (9) | 42 (9) | 0.057 | 0.009 | 313 (9) | 66 (9) | 0.031 | 0.017 |
| stroke | 628 (31) | 122 (30) | 0.104 | 0.021 | 656 (29) | 130 (29) | 0.163 | 0.006 | 1,100 (30) | 218 (30) | 0.067 | 0.006 |
| thromboembolism | 179 (9) | 37 (9) | 0.172 | 0.011 | 83 (4) | 21 (5) | 0.031 | 0.044 | 310 (8) | 60 (8) | 0.118 | 0.011 |
| arterial diseases**^*^** | 370 (18) | 70 (17) | 0.028 | 0.027 | 470 (21) | 97 (21) | 0.05 | 0.016 | 704 (19) | 141 (19) | 0.055 | <0.001 |
| heart failure | 1,001 (49) | 204 (50) | 0.051 | 0.023 | 1,149 (50) | 230 (50) | 0.142 | <0.001 | 1,855 (51) | 365 (50) | 0.087 | 0.016 |
| CKD | 199 (10) | 39 (10) | 0.157 | 0.008 | 72 (3) | 15 (3) | 0.014 | 0.007 | 194 (5) | 36 (5) | 0.066 | 0.019 |
| CHA2DS2-VASc |  |  |  |  |  |  |  |  |  |  |  |  |
| 0-1, n(%) | 265 (13) | 53 (13) | 0.085 | 0.001 | 300 (13) | 60 (13) | 0.009 | <0.001 | 428 (12) | 84 (11) | 0.112 | 0.006 |
| 2-3, n(%) | 618 (30) | 121 (30) | 0.147 | 0.015 | 833 (37) | 162 (36) | 0.034 | 0.022 | 1,358 (37) | 273 (37) | 0.077 | 0.004 |
| ≥4, n(%) | 1,147 (57) | 233 (57) | 0.084 | 0.015 | 1,144 (50) | 234 (51) | 0.039 | 0.022 | 1,869 (51) | 374 (51) | 0.152 | <0.001 |
| Medications, n(%) |  |  |  |  |  |  |  |  |  |  |  |  |
| low dosing NOAC | 1,026 (51) | 211 (52) | 0.022 | 0.026 | 1,421 (62) | 285 (63) | 0.014 | 0.002 | 1,480 (40) | 300 (41) | 0.005 | 0.011 |
| antiplatelet agent | 758 (37) | 152 (37) | 0.012 | 0.007 | 780 (34) | 157 (34) | 0.042 | 0.003 | 1,394 (38) | 280 (38) | 0.05 | 0.003 |
| statin | 1,085 (53) | 214 (53) | 0.025 | 0.018 | 1,177 (52) | 242 (53) | 0.206 | 0.028 | 1,824 (50) | 368 (50) | 0.164 | 0.009 |
| ACEI/ARB | 1,158 (57) | 226 (56) | 0.015 | 0.012 | 1,260 (55) | 243 (53) | 0.064 | 0.041 | 2,104 (58) | 411 (56) | 0.027 | 0.027 |
| beta blocker | 1,082 (53) | 211 (52) | 0.205 | 0.018 | 1,136 (50) | 224 (49) | 0.022 | 0.015 | 1,762 (48) | 356 (49) | 0.023 | 0.010 |
| CCB | 872 (43) | 170 (42) | 0.035 | 0.031 | 946 (42) | 189 (41) | 0.006 | 0.002 | 1,481 (41) | 291(40) | 0.036 | 0.014 |
| diuretics | 241 (12) | 51(13) | 0.025 | 0.029 | 259 (11) | 55 (12) | 0.102 | 0.022 | 447 (12) | 93 (13) | 0.096 | 0.016 |

PDC, proportion of days covered; NOAC, non-vitamin K antagonist oral anticoagulant; Edox/Riva, edoxaban or rivaroxaban; *d_before_*, standardized difference before propensity score matching; *d_after_*, standardized difference after propensity score matching; **^*^**stenosis or thrombosis of aortic and peripheral arteries; CKD, chronic kidney disease; ACEI/ARB, angiotensin-converting enzyme inhibitor or angiotensin-receptor blocker; CCB, calcium channel blocker
