# Supplementary material for: Adherence and clinical outcomes for twice-daily versus once-daily dosing of non-vitamin K antagonist oral anticoagulants in patients with atrial fibrillation: Is dosing frequency important?
Source: PLoS One. 2023 Mar 30;18(3):e0283478. doi: 10.1371/journal.pone.0283478 (PMC10062560; doi:10.1371/journal.pone.0283478)
Supplement: S4 Table — (DOCX) [file pone.0283478.s004.docx]

S4 Table. Baseline characteristics and standardized differences after propensity score matching for stroke, AMI, and death between patients with high (PDC ≥80%) and low (PDC <80%) adherence to each NOAC

| **Characteristics** | **Total** | | | | | | | | | | | |
| --- | --- | --- | --- | --- | --- | --- | --- | --- | --- | --- | --- | --- |
|  | **Stroke** | | | | **AMI** | | | | **Death** | | | |
|  | PDC ≥80% | PDC <80% | *d_before_* | *d_after_* | PDC ≥80% | PDC <80% | *d_before_* | *d_after_* | PDC ≥80% | PDC <80% | *d_before_* | *d_after_* |
| Subjects, n | 7,935 | 1,587 |  |  | 7,695 | 1,539 |  |  | 7,660 | 1,532 |  |  |
| Age, years | 73 ± 11 | 73 ± 13 | 0.022 | 0.015 | 72 ± 11 | 72 ± 13 | 0.016 | 0.020 | 72 ± 11 | 72 ± 13 | 0.013 | 0.019 |
| Male, n(%) | 4,067 (51) | 832 (52) | 0.003 | 0.023 | 4,163 (54) | 820 (53) | 0.014 | 0.016 | 4,113 (54) | 820 (54) | 0.019 | 0.003 |
| Medical history, n(%) |  |  |  |  |  |  |  |  |  |  |  |  |
| hypertension | 6,192 (78) | 1,223 (77) | 0.081 | 0.024 | 5,847 (76) | 1,181 (77) | 0.087 | 0.018 | 5,859 (76) | 1,175 (77) | 0.088 | 0.005 |
| diabetes | 1,806 (23) | 359 (23) | 0.070 | 0.003 | 1,682 (22) | 355 (23) | 0.057 | 0.028 | 1,709 (22) | 354 (23) | 0.056 | 0.019 |
| dyslipidemia | 3,897 (49) | 776 (49) | 0.225 | 0.004 | 3,791 (49) | 762 (50) | 0.210 | 0.005 | 3,786 (49) | 758 (49) | 0.211 | 0.001 |
| myocardial infarction | 786 (10) | 155 (10) | 0.037 | 0.005 | 759 (10) | 155 (10) | 0.050 | 0.007 | 780 (10) | 154 (10) | 0.051 | 0.004 |
| stroke | 2,307 (29) | 467 (29) | 0.039 | 0.008 | 2,212 (29) | 439 (29) | 0.051 | 0.005 | 2,146 (28) | 436 (28) | 0.052 | 0.010 |
| thromboembolism | 595 (7) | 116 (7) | 0.091 | 0.008 | 546 (7) | 115 (7) | 0.097 | 0.016 | 558 (7) | 113 (7) | 0.092 | 0.004 |
| arterial diseases**^*^** | 1,522 (19) | 307 (19) | 0.036 | 0.004 | 1,408 (18) | 287 (19) | 0.017 | 0.009 | 1,340 (17) | 286 (19) | 0.018 | 0.030 |
| heart failure | 4,024 (51) | 794 (50) | 0.087 | 0.014 | 3,723 (48) | 769 (50) | 0.088 | 0.032 | 3,798 (50) | 764 (50) | 0.086 | 0.006 |
| CKD | 483 (6) | 91 (6) | 0.079 | 0.016 | 429 (6) | 89 (6) | 0.083 | 0.010 | 445 (6) | 90 (6) | 0.087 | 0.03 |
| CHA2DS2-VASc |  |  |  |  |  |  |  |  |  |  |  |  |
| 0-1, n(%) | 1,007 (13) | 198 (12) | 0.027 | 0.006 | 991 (13) | 193 (13) | 0.027 | 0.010 | 948 (12) | 194 (13) | 0.024 | 0.003 |
| 2-3, n(%) | 2,735 (34) | 554 (35) | 0.083 | 0.009 | 2,787 (36) | 544 (35) | 0.074 | 0.018 | 2,791 (36) | 542 (35) | 0.073 | 0.017 |
| ≥4, n(%) | 4,193 (53) | 835 (53) | 0.099 | 0.005 | 3,917 (51) | 802 (52) | 0.090 | 0.024 | 3,921 (51) | 796 (52) | 0.087 | 0.014 |
| Medications, n(%) |  |  |  |  |  |  |  |  |  |  |  |  |
| low dosing NOAC | 3,989 (50) | 795 (50) | 0.031 | 0.004 | 3,708 (48) | 762 (50) | 0.021 | 0.027 | 3,818 (50) | 761 (50) | 0.024 | 0.003 |
| antiplatelet agent | 3,015 (38) | 588 (37) | 0.033 | 0.019 | 2,838 (37) | 568 (37) | 0.038 | <0.001 | 2,913 (38) | 567 (37) | 0.036 | 0.023 |
| statin | 4,120 (52) | 820 (52) | 0.183 | 0.005 | 4,015 (52) | 803 (52) | 0.170 | <0.001 | 3,977 (52) | 799 (52) | 0.170 | 0.005 |
| ACEI/ARB | 4,448 (56) | 876 (55) | 0.041 | 0.017 | 4,102 (53) | 838 (54) | 0.055 | 0.023 | 4,229 (55) | 834 (54) | 0.055 | 0.016 |
| beta blocker | 3,971 (50) | 790 (50) | 0.025 | 0.005 | 3,735 (49) | 757 (49) | 0.013 | 0.013 | 3,848 (50) | 756 (49) | 0.016 | 0.018 |
| CCB | 3,290 (41) | 647(41) | 0.031 | 0.014 | 2,967 (39) | 622 (40) | 0.038 | 0.038 | 3,056 (40) | 619 (40) | 0.038 | 0.010 |
| diuretics | 1,034 (13) | 200 (13) | 0.079 | 0.014 | 1,011 (13) | 197 (13) | 0.088 | 0.011 | 1,023 (13) | 198 (13) | 0.092 | 0.014 |
| **Characteristics** | **Apixaban** | | | | | | | | | | | |
|  | **Stroke** | | | | **AMI** | | | | **Death** | | | |
|  | PDC ≥80% | PDC <80% | *d_before_* | *d_after_* | PDC ≥80% | PDC <80% | *d_before_* | *d_after_* | PDC ≥80% | PDC <80% | *d_before_* | *d_after_* |
| Subjects, n | 2,021 | 405 |  |  | 1,985 | 397 |  |  | 1,975 | 395 |  |  |
| Age, years | 73 ± 11 | 73 ± 13 | 0.047 | 0.018 | 73 ± 11 | 73 ± 13 | 0.058 | 0.013 | 73 ± 12 | 73 ± 13 | 0.057 | 0.020 |
| Male, n(%) | 1,093 (54) | 215 (53) | 0.098 | 0.020 | 1,079 (54) | 216 (54) | 0.123 | 0.001 | 1,079 (55) | 216 (55) | 0.130 | 0.001 |
| Medical history, n(%) |  |  |  |  |  |  |  |  |  |  |  |  |
| hypertension | 1,605 (79) | 315 (78) | 0.070 | 0.040 | 1,528 (77) | 306 (77) | 0.086 | 0.002 | 1,536 (78) | 305 (77) | 0.082 | 0.014 |
| diabetes | 499 (25) | 96 (24) | 0.061 | 0.023 | 478 (24) | 98 (25) | 0.036 | 0.014 | 476 (24) | 98 (25) | 0.032 | 0.016 |
| dyslipidemia | 996 (49) | 194 (48) | 0.267 | 0.028 | 952 (48) | 191 (48) | 0.263 | 0.003 | 966 (49) | 190 (48) | 0.263 | 0.016 |
| myocardial infarction | 242 (12) | 49 (12) | 0.056 | 0.004 | 207 (10) | 46 (12) | 0.041 | 0.037 | 242 (12) | 47 (12) | 0.054 | 0.011 |
| stroke | 616 (30) | 122 (30) | 0.101 | 0.008 | 572 (29) | 113 (28) | 0.130 | 0.008 | 557 (28) | 113 (29) | 0.127 | 0.009 |
| thromboembolism | 180 (9) | 37 (9) | 0.173 | 0.009 | 177 (9) | 34 (9) | 0.153 | 0.014 | 164 (8) | 34 (9) | 0.154 | 0.012 |
| arterial diseases**^*^** | 337 (17) | 69 (17) | 0.033 | 0.010 | 332 (17) | 68 (17) | 0.030 | 0.011 | 318 (16) | 67 (17) | 0.035 | 0.023 |
| heart failure | 1,021 (51) | 205 (51) | 0.061 | 0.002 | 1,026 (52) | 200 (50) | 0.058 | 0.026 | 997 (50) | 201 (51) | 0.068 | 0.008 |
| CKD | 204 (10) | 40 (10) | 0.167 | 0.008 | 185 (9) | 37 (9) | 0.150 | <0.001 | 210 (11) | 38 (10) | 0.161 | 0.039 |
| CHA2DS2-VASc |  |  |  |  |  |  |  |  |  |  |  |  |
| 0-1, n(%) | 283 (14) | 53 (13) | 0.087 | 0.029 | 270 (14) | 53 (13) | 0.092 | 0.008 | 284 (14) | 53 (13) | 0.068 | 0.008 |
| 2-3, n(%) | 594 (29) | 119 (29) | 0.155 | <0.001 | 605 (30) | 120 (30) | 0.136 | 0.005 | 588 (30) | 118 (30) | 0.094 | 0.030 |
| ≥4, n(%) | 1,144 (57) | 233 (58) | 0.091 | 0.019 | 1110 (56) | 224 (56) | 0.069 | 0.010 | 1,103 (56) | 224 (57) | 0.145 | 0.002 |
| Medications, n(%) |  |  |  |  |  |  |  |  |  |  |  |  |
| low dosing NOAC | 1,053 (52) | 212 (52) | 0.032 | 0.005 | 1,057 (53) | 208 (52) | 0.037 | 0.017 | 1,018 (52) | 209 (53) | 0.048 | 0.027 |
| antiplatelet agent | 752 (37) | 151 (37) | 0.010 | 0.002 | 745 (38) | 151 (38) | 0.024 | 0.010 | 762 (39) | 150 (38) | 0.022 | 0.013 |
| statin | 1,081 (53) | 213 (53) | 0.204 | 0.018 | 1,032 (52) | 207 (52) | 0.213 | 0.003 | 1,044 (53) | 206 (52) | 0.212 | 0.014 |
| ACEI/ARB | 1,164 (58) | 225 (56) | 0.035 | 0.041 | 1,112 (56) | 216 (54) | 0.056 | 0.032 | 1,073 (54) | 215 (54) | 0.055 | 0.002 |
| beta blocker | 1,120 (55) | 212 (52) | 0.036 | 0.061 | 1,051 (53) | 208 (52) | 0.037 | 0.011 | 1,054 (53) | 209 (53) | 0.048 | 0.009 |
| CCB | 861 (43) | 169 (42) | 0.043 | 0.018 | 815 (41) | 158 (40) | 0.083 | 0.026 | 752 (38) | 157 (40) | 0.084 | 0.034 |
| diuretics | 266 (13) | 52 (13) | 0.041 | 0.010 | 265 (13) | 51 (13) | 0.045 | 0.015 | 262 (13) | 52 (13) | 0.055 | 0.003 |
| **Characteristics** | **Dabigatran** | | | | | | | | | | | |
|  | **Stroke** | | | | **AMI** | | | | **Death** | | | |
|  | PDC ≥80% | PDC <80% | *d_before_* | *d_after_* | PDC ≥80% | PDC <80% | *d_before_* | *d_after_* | PDC ≥80% | PDC <80% | *d_before_* | *d_after_* |
| Subjects, n | 2,272 | 455 |  |  | 2,193 | 439 |  |  | 2,188 | 438 |  |  |
| Age, years | 71 ± 11 | 71 ± 13 | 0.01 | 0.012 | 71 ± 11 | 71 ± 13 | 0.003 | 0.019 | 71 ± 11 | 71 ± 13 | 0.006 | 0.014 |
| Male, n(%) | 1,155 (51) | 238 (52) | 0.08 | 0.03 | 1,166 (53) | 230 (52) | 0.08 | 0.016 | 1,122 (51) | 230 (53) | 0.077 | 0.025 |
| Medical history, n(%) |  |  |  |  |  |  |  |  |  |  |  |  |
| hypertension | 1,726 (76) | 344 (76) | 0.095 | 0.009 | 1,650 (75) | 333 (76) | 0.087 | 0.015 | 1,661 (76) | 332 (76) | 0.088 | 0.003 |
| diabetes | 497 (22) | 103 (23) | 0.063 | 0.018 | 464 (21) | 97 (22) | 0.074 | 0.022 | 499 (23) | 97 (22) | 0.072 | 0.016 |
| dyslipidemia | 1,162 (51) | 230 (51) | 0.242 | 0.012 | 1,131 (52) | 227 (52) | 0.216 | 0.003 | 1,134 (52) | 227 (52) | 0.213 | <0.001 |
| myocardial infarction | 184 (8) | 42 (9) | 0.059 | 0.041 | 210 (10) | 44 (10) | 0.09 | 0.016 | 231 (11) | 44 (10) | 0.093 | 0.018 |
| stroke | 648 (29) | 129 (28) | 0.167 | 0.004 | 626 (29) | 124 (28) | 0.16 | 0.006 | 624 (29) | 123 (28) | 0.164 | 0.009 |
| thromboembolism | 106 (5) | 21 (5) | 0.030 | 0.002 | 111 (5) | 22 (5) | 0.012 | 0.002 | 107 (5) | 22 (5) | 0.011 | 0.006 |
| arterial diseases**^*^** | 488 (21) | 96 (21) | 0.045 | 0.009 | 449 (20) | 92 (21) | 0.042 | 0.012 | 457 (21) | 91 (21) | 0.036 | 0.003 |
| heart failure | 1,144 (50) | 229 (50) | 0.14 | <0.001 | 1,101 (50) | 222 (51) | 0.153 | 0.008 | 1,099 (50) | 222 (51) | 0.151 | 0.006 |
| CKD | 69 (3) | 15 (3) | 0.013 | 0.015 | 73 (3) | 16 (4) | 0.033 | 0.018 | 75 (3) | 16 (4) | 0.032 | 0.012 |
| CHA2DS2-VASc |  |  |  |  |  |  |  |  |  |  |  |  |
| 0-1, n(%) | 330 (15) | 60 (13) | 0.008 | 0.039 | 281 (13) | 57(13) | 0.017 | 0.005 | 317 (14) | 57 (13) | 0.016 | 0.043 |
| 2-3, n(%) | 830 (37) | 162 (36) | 0.033 | 0.019 | 768 (35) | 154(35) | 0.045 | 0.002 | 763 (35) | 154 (35) | 0.044 | 0.007 |
| ≥4, n(%) | 1,112 (49) | 233 (51) | 0.037 | 0.045 | 1,144 (52) | 228 (52) | 0.055 | 0.005 | 1,108 (51) | 227 (52) | 0.053 | 0.023 |
| Medications, n(%) |  |  |  |  |  |  |  |  |  |  |  |  |
| low dosing NOAC | 1,441 (63) | 284 (62) | 0.012 | 0.021 | 1,387 (63) | 274 (62) | 0.011 | 0.017 | 1,357 (62) | 273 (62) | 0.010 | 0.006 |
| antiplatelet agent | 803 (35) | 156 (34) | 0.046 | 0.022 | 724 (33) | 149 (34) | 0.054 | 0.02 | 735 (34) | 148 (34) | 0.058 | 0.005 |
| statin | 1,212 (53) | 242 (53) | 0.203 | 0.003 | 1,180 (54) | 238 (54) | 0.18 | 0.008 | 1,179 (54) | 238 (54) | 0.177 | 0.009 |
| ACEI/ARB | 1,192 (52) | 243 (53) | 0.061 | 0.019 | 1,183 (54) | 235 (54) | 0.058 | 0.008 | 1,174 (54) | 235 (54) | 0.054 | <0.001 |
| beta blocker | 1,131 (50) | 223 (49) | 0.021 | 0.015 | 1,049 (48) | 211 (48) | <0.001 | 0.005 | 1,047 (48) | 210 (48) | 0.002 | 0.002 |
| CCB | 940 (41) | 188 (41) | 0.008 | 0.001 | 921 (42) | 185 (42) | 0.009 | 0.003 | 924 (42) | 184 (42) | 0.007 | 0.004 |
| diuretics | 273 (12) | 55 (12) | 0.103 | 0.002 | 264 (12) | 55 (13) | 0.119 | 0.016 | 247 (11) | 55 (13) | 0.120 | 0.041 |
| **Characteristics** | **Edox/Riva** | | | | | | | | | | | |
|  | **Stroke** | | | | **AMI** | | | | **Death** | | | |
|  | PDC ≥80% | PDC <80% | *d_before_* | *d_after_* | PDC ≥80% | PDC <80% | *d_before_* | *d_after_* | PDC ≥80% | PDC <80% | *d_before_* | *d_after_* |
| Subjects, n | 3,635 | 727 |  |  | 3515 | 703 |  |  | 3495 | 699 |  |  |
| Age, years | 73 ± 11 | 73 ± 13 | 0.103 | 0.023 | 73 ± 11 | 73 ± 14 | 0.088 | 0.021 | 73 ± 11 | 73 ± 14 | 0.084 | 0.019 |
| Male, n(%) | 1,905 (52) | 379 (52) | 0.030 | 0.006 | 1,966 (56) | 374 (53) | 0.008 | 0.055 | 1,884 (54) | 374 (54) | 0.002 | 0.008 |
| Medical history, n(%) |  |  |  |  |  |  |  |  |  |  |  |  |
| hypertension | 2,812 (77) | 564 (78) | 0.073 | 0.005 | 2,723 (77) | 542 (77) | 0.083 | 0.009 | 2,681 (77) | 538 (77) | 0.086 | 0.006 |
| diabetes | 823 (23) | 160 (22) | 0.077 | 0.015 | 782 (22) | 160 (23) | 0.057 | 0.012 | 780 (22) | 159 (23) | 0.057 | 0.01 |
| dyslipidemia | 1,740 (48) | 352 (48) | 0.2 | 0.011 | 1,693 (48) | 344 (49) | 0.185 | 0.015 | 1,718 (49) | 341 (49) | 0.188 | 0.007 |
| myocardial infarction | 327 (9) | 64 (9) | 0.024 | 0.007 | 347 (10) | 65 (9) | 0.041 | 0.022 | 307 (9) | 63 (9) | 0.033 | 0.008 |
| stroke | 1,074 (30) | 216 (30) | 0.065 | 0.004 | 970 (28) | 202 (29) | 0.051 | 0.025 | 1,000 (29) | 200 (29) | 0.048 | <0.001 |
| thromboembolism | 300 (8) | 58 (8) | 0.109 | 0.011 | 297 (8) | 59 (8) | 0.123 | 0.002 | 285 (8) | 57 (8) | 0.114 | <0.001 |
| arterial diseases**^*^** | 722 (20) | 142 (20) | 0.061 | 0.009 | 614 (17) | 127 (18) | 0.023 | 0.016 | 625 (18) | 128 (18) | 0.029 | 0.011 |
| heart failure | 1,790 (49) | 360 (50) | 0.061 | 0.002 | 1,691 (48) | 346 (49) | 0.075 | 0.022 | 1,661 (47) | 341 (49) | 0.067 | 0.025 |
| CKD | 184 (5) | 36 (5) | 0.068 | 0.005 | 177 (5) | 36 (5) | 0.077 | 0.004 | 205 (6) | 36 (5) | 0.079 | 0.035 |
| CHA2DS2-VASc |  |  |  |  |  |  |  |  |  |  |  |  |
| 0-1, n(%) | 437 (12) | 85 (12) | 0.106 | 0.01 | 398 (11) | 83 (12) | 0.103 | 0.014 | 435 (12) | 84 (12) | 0.097 | 0.013 |
| 2-3, n(%) | 1,349 (37) | 273 (38) | 0.073 | 0.009 | 1,400 (40) | 270 (38) | 0.054 | 0.029 | 1,371 (39) | 270 (39) | 0.049 | 0.012 |
| ≥4, n(%) | 1,849 (51) | 369 (51) | 0.144 | 0.002 | 1,717 (49) | 350 (50) | 0.124 | 0.019 | 1,689 (48) | 345 (49) | 0.115 | 0.021 |
| Medications, n(%) |  |  |  |  |  |  |  |  |  |  |  |  |
| low dosing NOAC | 1,487 (41) | 299 (41) | 0.003 | 0.004 | 1,365 (39) | 280 (40) | 0.029 | 0.020 | 1,370 (39) | 279 (40) | 0.027 | 0.015 |
| antiplatelet agent | 1,379 (38) | 281 (39) | 0.043 | 0.015 | 1,323 (38) | 268 (38) | 0.057 | 0.010 | 1,377 (39) | 269 (38) | 0.050 | 0.019 |
| statin | 1,816 (50) | 365 (50) | 0.166 | 0.005 | 1,753 (50) | 358 (51) | 0.147 | 0.021 | 1,787 (51) | 355 (51) | 0.150 | 0.007 |
| ACEI/ARB | 2,024 (56) | 408 (56) | 0.029 | 0.009 | 1,961 (56) | 387 (55) | 0.049 | 0.015 | 1,948 (56) | 384 (55) | 0.052 | 0.016 |
| beta blocker | 1,753 (48) | 355 (49) | 0.026 | 0.012 | 1,674 (48) | 338 (48) | 0.011 | 0.009 | 1,623(46) | 337 (48) | 0.014 | 0.036 |
| CCB | 1,450 (40) | 290 (40) | 0.035 | <0.001 | 1,388 (39) | 279 (40) | 0.038 | 0.004 | 1,377 (39) | 278 (40) | 0.036 | 0.008 |
| diuretics | 477 (13) | 93 (13) | 0.098 | 0.01 | 459 (13) | 91 (13) | 0.103 | 0.004 | 453 (13) | 91 (13) | 0.106 | 0.002 |

AMI, acute myocardial infarction; NOAC, non-vitamin K antagonist oral anticoagulant; PDC, proportion of days covered; *d_before_*, standardized difference before propensity score matching; *d_after_*, standardized difference after propensity score matching; **^*^**stenosis or thrombosis of aortic and peripheral arteries; CKD, chronic kidney disease; ACEI/ARB, angiotensin-converting enzyme inhibitor or angiotensin-receptor blocker; CCB, calcium channel blocker; Edox/Riva, edoxaban or rivaroxaban
