# Supplementary material for: Adherence and clinical outcomes for twice-daily versus once-daily dosing of non-vitamin K antagonist oral anticoagulants in patients with atrial fibrillation: Is dosing frequency important?
Source: PLoS One. 2023 Mar 30;18(3):e0283478. doi: 10.1371/journal.pone.0283478 (PMC10062560; doi:10.1371/journal.pone.0283478)
Supplement: S5 Table — (DOCX) [file pone.0283478.s005.docx]

S5 Table. Baseline characteristics and standardized differences after propensity score matching for composite outcome, stroke, AMI, and death between NOAC classes

| **A. Twice-daily regimen vs. Once-daily regimen** | | | | | | | | | | | | | | | | | | | | | | | | | | | | | | | | | | | | | |
| --- | --- | --- | --- | --- | --- | --- | --- | --- | --- | --- | --- | --- | --- | --- | --- | --- | --- | --- | --- | --- | --- | --- | --- | --- | --- | --- | --- | --- | --- | --- | --- | --- | --- | --- | --- | --- | --- |
| **Characteristics** | **Composite outcome** | | | | | | | | | | | | | | | | | | | | | | | | | | | | | | | | | | | | |
|  | **High adherence group** | | | | | | | | | | | | | | | | | | | | | | | | | **Low adherence group** | | | | | | | | | | | |
|  | Twice-daily | Once-daily | | | | | | | | *d_before_* | | | | | | | | *d_after_* | | | | | | | | Twice-daily | | | Once-daily | | | *d_before_* | | | *d_after_* | | |
| Subjects, n | 15,140 | 15,140 | | | | | | | |  | | | | | | | |  | | | | | | | | 698 | | | 698 | | |  | | |  | | |
| Age, years | 72 ± 11 | 72 ± 11 | | | | | | | | 0.065 | | | | | | | | 0.012 | | | | | | | | 72 ± 13 | | | 73 ± 13 | | | 0.074 | | | 0.045 | | |
| Male, n(%) | 7,936 (52) | 7,980 (53) | | | | | | | | 0.043 | | | | | | | | 0.006 | | | | | | | | 371 (53) | | | 367 (53) | | | <0.001 | | | 0.012 | | |
| Medical history, n(%) |  |  | | | | | | | |  | | | | | | | |  | | | | | | | |  | | |  | | |  | | |  | | |
| hypertension | 12,149 (80) | 12,105 (80) | | | | | | | | 0.007 | | | | | | | | 0.007 | | | | | | | | 531 (76) | | | 540 (77) | | | 0.013 | | | 0.031 | | |
| diabetes | 4,027 (27) | 3,858 (25) | | | | | | | | 0.015 | | | | | | | | 0.026 | | | | | | | | 157 (22) | | | 154 (22) | | | 0.015 | | | 0.01 | | |
| dyslipidemia | 9,230 (61) | 8,984 (59) | | | | | | | | 0.072 | | | | | | | | 0.033 | | | | | | | | 343 (49) | | | 339 (49) | | | 0.024 | | | 0.011 | | |
| myocardial infarction | 1,409 (9) | 1,282 (8) | | | | | | | | 0.039 | | | | | | | | 0.03 | | | | | | | | 70 (10) | | | 62 (9) | | | 0.06 | | | 0.037 | | |
| stroke | 4,974 (33) | 4,422 (29) | | | | | | | | 0.188 | | | | | | | | 0.079 | | | | | | | | 200 (29) | | | 202 (29) | | | 0.007 | | | 0.006 | | |
| thromboembolism | 761 (5) | 782 (5) | | | | | | | | 0.020 | | | | | | | | 0.006 | | | | | | | | 49 (7) | | | 51 (7) | | | 0.058 | | | 0.011 | | |
| arterial diseases**^*^** | 2,779 (18) | 2,647 (17) | | | | | | | | 0.042 | | | | | | | | 0.023 | | | | | | | | 131 (19) | | | 133 (19) | | | 0.021 | | | 0.007 | | |
| heart failure | 6,993 (46) | 7,030 (46) | | | | | | | | 0.005 | | | | | | | | 0.009 | | | | | | | | 344 (49) | | | 347 (50) | | | 0.029 | | | 0.009 | | |
| CKD | 694 (5) | 565 (4) | | | | | | | | 0.048 | | | | | | | | 0.044 | | | | | | | | 38 (5) | | | 36 (5) | | | 0.055 | | | 0.012 | | |
| CHA2DS2-VASc |  |  | | | | | | | |  | | | | | | | |  | | | | | | | |  | | |  | | |  | | |  | | |
| 0-1, n(%) | 1,891 (12) | 2,002 (13) | | | | | | | | 0.109 | | | | | | | | 0.026 | | | | | | | | 103 (15) | | | 84 (12) | | | 0.04 | | | 0.058 | | |
| 2-3, n(%) | 5,670 (37) | 6,133 (41) | | | | | | | | 0.087 | | | | | | | | 0.061 | | | | | | | | 232 (33) | | | 259 (37) | | | 0.115 | | | 0.082 | | |
| ≥4, n(%) | 7,579 (50) | 7,005 (46) | | | | | | | | 0.159 | | | | | | | | 0.077 | | | | | | | | 363 (52) | | | 355 (51) | | | 0.084 | | | 0.024 | | |
| Medications, n(%) |  |  | | | | | | | |  | | | | | | | |  | | | | | | | |  | | |  | | |  | | |  | | |
| antiplatelet agent | 5,644 (37) | 5,994 (40) | | | | | | | | 0.084 | | | | | | | | 0.045 | | | | | | | | 249 (36) | | | 263 (38) | | | 0.054 | | | 0.043 | | |
| statin | 9,401 (62) | 9,064 (60) | | | | | | | | 0.094 | | | | | | | | 0.046 | | | | | | | | 362 (52) | | | 354 (51) | | | 0.049 | | | 0.024 | | |
| ACEI/ARB | 8,638 (57) | 8,589 (57) | | | | | | | | 0.009 | | | | | | | | 0.007 | | | | | | | | 384 (55) | | | 388 (56) | | | 0.019 | | | 0.012 | | |
| beta blocker | 7,606 (50) | 7,388 (49) | | | | | | | | 0.04 | | | | | | | | 0.029 | | | | | | | | 350 (50) | | | 346 (50) | | | 0.042 | | | 0.012 | | |
| CCB | 6,500 (43) | 6,291 (42) | | | | | | | | 0.032 | | | | | | | | 0.028 | | | | | | | | 283 (41) | | | 276 (40) | | | 0.025 | | | 0.021 | | |
| Diuretics | 1,571 (10) | 1,507 (10) | | | | | | | | 0.058 | | | | | | | | 0.007 | | | | | | | | 80 (11) | | | 82(12) | | | 0.006 | | | 0.006 | | |
| **Characteristics** | **Stroke** | | | | | | | | | | | | | | | | | | | | | | | | | | | | | | | | | | | | |
|  | **High adherence group** | | | | | | | | | | | | | | | | | | | | | | | | | **Low adherence group** | | | | | | | | | | | |
|  | Twice-daily | Once-daily | | | | | | | | *d_before_* | | | | | | | | *d_after_* | | | | | | | | Twice-daily | | | Once-daily | | | *d_before_* | | | *d_after_* | | |
| Subjects, n | 14,152 | 14,152 | | | | | | | |  | | | | | | | |  | | | | | | | | 693 | | | 693 | | |  | | |  | | |
| Age, years | 72 ± 11 | 72 ± 11 | | | | | | | | 0.087 | | | | | | | | 0.017 | | | | | | | | 73 ± 13 | | | 72 ± 12 | | | 0.073 | | | 0.022 | | |
| Male, n(%) | 7,520 (53) | 7,443 (53) | | | | | | | | 0.011 | | | | | | | | 0.014 | | | | | | | | 368 (53) | | | 363 (52) | | | <0.001 | | | 0.018 | | |
| Medical history, n(%) |  |  | | | | | | | |  | | | | | | | |  | | | | | | | |  | | |  | | |  | | |  | | |
| hypertension | 11,339 (80) | 11,326 (80) | | | | | | | | 0.023 | | | | | | | | 0.014 | | | | | | | | 535 (77) | | | 539 (78) | | | 0.012 | | | <0.001 | | |
| diabetes | 3,589 (25) | 3,712 (26) | | | | | | | | 0.027 | | | | | | | | 0.017 | | | | | | | | 155 (22) | | | 160 (23) | | | 0.016 | | | 0.004 | | |
| dyslipidemia | 8,397 (59) | 8,682 (61) | | | | | | | | 0.018 | | | | | | | | <0.001 | | | | | | | | 341 (49) | | | 341 (49) | | | 0.026 | | | 0.040 | | |
| myocardial infarction | 1,197 (8) | 1,293 (9) | | | | | | | | 0.060 | | | | | | | | 0.044 | | | | | | | | 60 (9) | | | 69 (10) | | | 0.064 | | | 0.026 | | |
| stroke | 4,136 (29) | 4,614 (33) | | | | | | | | 0.012 | | | | | | | | 0.028 | | | | | | | | 199 (29) | | | 208 (30) | | | 0.006 | | | 0.027 | | |
| thromboembolism | 713 (5) | 716 (5) | | | | | | | | 0.047 | | | | | | | | 0.017 | | | | | | | | 51 (7) | | | 48 (7) | | | 0.055 | | | 0.012 | | |
| arterial diseases**^*^** | 2,448 (17) | 2,590 (18) | | | | | | | | 0.009 | | | | | | | | 0.018 | | | | | | | | 134 (19) | | | 129 (19) | | | 0.017 | | | 0.008 | | |
| heart failure | 6,507 (46) | 6,478 (46) | | | | | | | | 0.004 | | | | | | | | 0.004 | | | | | | | | 344 (50) | | | 336 (48) | | | 0.019 | | | 0.024 | | |
| CKD | 538 (4) | 632 (4) | | | | | | | | 0.062 | | | | | | | | 0.025 | | | | | | | | 36 (5) | | | 40 (6) | | | 0.057 | | | 0.033 | | |
| CHA2DS2-VASc |  |  | | | | | | | |  | | | | | | | |  | | | | | | | |  | | |  | | |  | | |  | | |
| 0-1, n(%) | 1,760 (12) | 1,891 (13) | | | | | | | | 0.108 | | | | | | | | 0.027 | | | | | | | | 91 (13) | | | 85 (12) | | | 0.044 | | | 0.027 | | |
| 2-3, n(%) | 5,301 (37) | 5,682 (40) | | | | | | | | 0.086 | | | | | | | | 0.055 | | | | | | | | 224 (32) | | | 245 (35) | | | 0.102 | | | 0.065 | | |
| ≥4, n(%) | 7,091 (50) | 6,579 (46) | | | | | | | | 0.159 | | | | | | | | 0.073 | | | | | | | | 378 (55) | | | 363 (52) | | | 0.069 | | | 0.044 | | |
| Medications, n(%) |  |  | | | | | | | |  | | | | | | | |  | | | | | | | |  | | |  | | |  | | |  | | |
| antiplatelet agent | 5,320 (38) | 5,625 (40) | | | | | | | | 0.084 | | | | | | | | 0.044 | | | | | | | | 250 (36) | | | 258 (37) | | | 0.061 | | | 0.024 | | |
| statin | 8,468 (60) | 8,836 (62) | | | | | | | | 0.054 | | | | | | | | 0.02 | | | | | | | | 355 (51) | | | 362 (52) | | | 0.05 | | | 0.025 | | |
| ACEI/ARB | 8,071 (57) | 8,015 (57) | | | | | | | | 0.034 | | | | | | | | 0.006 | | | | | | | | 384 (55) | | | 386 (56) | | | 0.018 | | | 0.028 | | |
| beta blocker | 6,943 (49) | 7,106 (50) | | | | | | | | 0.035 | | | | | | | | 0.003 | | | | | | | | 343 (49) | | | 344 (50) | | | 0.042 | | | 0.006 | | |
| CCB | 5,940 (42) | 6,026 (43) | | | | | | | | 0.033 | | | | | | | | 0.015 | | | | | | | | 276 (40) | | | 281 (41) | | | 0.024 | | | 0.019 | | |
| Diuretics | 1,409 (10) | 1,499 (11) | | | | | | | | 0.011 | | | | | | | | 0.03 | | | | | | | | 84 (12) | | | 91 (13) | | | 0.005 | | | 0.009 | | |
| **Characteristics** | **AMI** | | | | | | | | | | | | | | | | | | | | | | | | | | | | | | | | | | | | |
|  | **High adherence group** | | | | | | | | | | | | | | | | | | | | | | | | | | **Low adherence group** | | | | | | | | | | |
|  | Twice-daily | | Once-daily | | | | | | | | *d_before_* | | | | | | | | *d_after_* | | | | | | | | Twice-daily | | | Once-daily | | | *d_before_* | | | *d_after_* | |
| Subjects, n | 14,177 | | 14,177 | | | | | | | |  | | | | | | | |  | | | | | | | | 659 | | | 659 | | |  | | |  | |
| Age, years | 72 ± 11 | | 72 ± 11 | | | | | | | | 0.061 | | | | | | | | 0.014 | | | | | | | | 72 ± 14 | | | 72 ± 13 | | | 0.076 | | | 0.048 | |
| Male, n(%) | 7,504 (53) | | 7,495 (53) | | | | | | | | 0.039 | | | | | | | | 0.001 | | | | | | | | 355 (54) | | | 355 (54) | | | 0.003 | | | <0.001 | |
| Medical history, n(%) |  | |  | | | | | | | |  | | | | | | | |  | | | | | | | |  | | |  | | |  | | |  | |
| hypertension | 11,362 (80) | | 11,373 (80) | | | | | | | | 0.009 | | | | | | | | 0.002 | | | | | | | | 505 (77) | | | 514 (78) | | | 0.016 | | | 0.032 | |
| diabetes | 3,608 (25) | | 3,684 (26) | | | | | | | | 0.015 | | | | | | | | 0.012 | | | | | | | | 149 (23) | | | 160 (24) | | | 0.013 | | | 0.04 | |
| dyslipidemia | 8,415 (59) | | 8,638 (61) | | | | | | | | 0.071 | | | | | | | | 0.032 | | | | | | | | 327 (50) | | | 324 (49) | | | 0.021 | | | 0.009 | |
| myocardial infarction | 1,185 (8) | | 1,278 (9) | | | | | | | | 0.036 | | | | | | | | 0.023 | | | | | | | | 60 (9) | | | 65 (10) | | | 0.051 | | | 0.025 | |
| stroke | 4,085 (29) | | 4,598 (32) | | | | | | | | 0.186 | | | | | | | | 0.079 | | | | | | | | 188 (29) | | | 189 (29) | | | 0.009 | | | 0.003 | |
| thromboembolism | 721 (5) | | 694 (5) | | | | | | | | 0.015 | | | | | | | | 0.009 | | | | | | | | 49 (7) | | | 48 (7) | | | 0.064 | | | 0.006 | |
| arterial diseases**^*^** | 2,470 (17) | | 2,577 (18) | | | | | | | | 0.039 | | | | | | | | 0.020 | | | | | | | | 121 (18) | | | 120 (18) | | | 0.028 | | | 0.004 | |
| heart failure | 6,564 (46) | | 6,473 (46) | | | | | | | | 0.005 | | | | | | | | 0.013 | | | | | | | | 331 (50) | | | 325 (49) | | | 0.028 | | | 0.018 | |
| CKD | 536 (4) | | 616 (4) | | | | | | | | 0.045 | | | | | | | | 0.029 | | | | | | | | 36 (5) | | | 35 (5) | | | 0.052 | | | 0.007 | |
| CHA2DS2-VASc |  | |  | | | | | | | |  | | | | | | | |  | | | | | | | |  | | |  | | |  | | |  | |
| 0-1, n(%) | 1,796 (13) | | 1,908 (13) | | | | | | | | 0.106 | | | | | | | | 0.023 | | | | | | | | 89 (14) | | | 83 (13) | | | 0.041 | | | 0.027 | |
| 2-3, n(%) | 5,279 (37) | | 5,676 (40) | | | | | | | | 0.085 | | | | | | | | 0.057 | | | | | | | | 237 (36) | | | 249 (38) | | | 0.118 | | | 0.038 | |
| ≥4, n(%) | 7,102 (50) | | 6,593 (47) | | | | | | | | 0.156 | | | | | | | | 0.072 | | | | | | | | 333 (51) | | | 327 (50) | | | 0.086 | | | 0.018 | |
| Medications, n(%) |  | |  | | | | | | | |  | | | | | | | |  | | | | | | | |  | | |  | | |  | | |  | |
| antiplatelet agent | 5,289 (37) | | 5,634 (40) | | | | | | | | 0.086 | | | | | | | | 0.050 | | | | | | | | 240 (36) | | | 249 (38) | | | 0.046 | | | 0.028 | |
| statin | 8,484 (60) | | 8,803 (62) | | | | | | | | 0.093 | | | | | | | | 0.046 | | | | | | | | 341 (52) | | | 345 (52) | | | 0.046 | | | 0.012 | |
| ACEI/ARB | 8,084 (57) | | 8,094 (57) | | | | | | | | 0.013 | | | | | | | | 0.001 | | | | | | | | 354 (54) | | | 355 (54) | | | 0.022 | | | 0.003 | |
| beta blocker | 6,933 (49) | | 7,123 (50) | | | | | | | | 0.040 | | | | | | | | 0.027 | | | | | | | | 324 (49) | | | 333 (51) | | | 0.041 | | | 0.027 | |
| CCB | 5,945 (42) | | 6,021 (42) | | | | | | | | 0.029 | | | | | | | | 0.011 | | | | | | | | 261 (40) | | | 268 (41) | | | 0.027 | | | 0.022 | |
| Diuretics | 1,399 (10) | | 1,452 (10) | | | | | | | | 0.021 | | | | | | | | 0.012 | | | | | | | | 76 (12) | | | 87 (13) | | | 0.008 | | | 0.050 | |
| **Characteristics** | **Death** | | | | | | | | | | | | | | | | | | | | | | | | | | | | | | | | | | | | |
|  | **High adherence group** | | | | | | | | | | | | | | | | | | | | | | | | | | **Low adherence group** | | | | | | | | | | |
|  | Twice-daily | | Once-daily | | | | | | | | *d_before_* | | | | | | | | *d_after_* | | | | | | | | Twice-daily | | | Once-daily | | | *d_before_* | | | *d_after_* | |
| Subjects, n | 14,171 | | 14,171 | | | | | | | |  | | | | | | | |  | | | | | | | | 652 | | | 652 | | |  | | |  | |
| Age, years | 72 ± 11 | | 72 ± 11 | | | | | | | | 0.060 | | | | | | | | 0.008 | | | | | | | | 72 ± 14 | | | 72 ± 13 | | | 0.073 | | | 0.022 | |
| Male, n(%) | 7,481 (53) | | 7,434 (52) | | | | | | | | 0.039 | | | | | | | | 0.007 | | | | | | | | 356 (55) | | | 350 (54) | | | <0.001 | | | 0.018 | |
| Medical history, n(%) |  | |  | | | | | | | |  | | | | | | | |  | | | | | | | |  | | |  | | |  | | |  | |
| hypertension | 11,335 (80) | | 11,350 (80) | | | | | | | | 0.010 | | | | | | | | 0.003 | | | | | | | | 498 (76) | | | 498 (76) | | | 0.012 | | | <0.001 | |
| diabetes | 3,578 (25) | | 3,706 (26) | | | | | | | | 0.014 | | | | | | | | 0.021 | | | | | | | | 151 (23) | | | 152 (23) | | | 0.016 | | | 0.004 | |
| dyslipidemia | 8,376 (59) | | 8,653 (61) | | | | | | | | 0.071 | | | | | | | | 0.040 | | | | | | | | 326 (50) | | | 313 (48) | | | 0.026 | | | 0.040 | |
| myocardial infarction | 1,175 (8) | | 1,264 (9) | | | | | | | | 0.035 | | | | | | | | 0.022 | | | | | | | | 59 (9) | | | 64 (10) | | | 0.064 | | | 0.026 | |
| stroke | 4,090 (29) | | 4,578 (32) | | | | | | | | 0.186 | | | | | | | | 0.075 | | | | | | | | 184 (28) | | | 176 (27) | | | 0.006 | | | 0.027 | |
| thromboembolism | 735 (5) | | 711 (5) | | | | | | | | 0.015 | | | | | | | | 0.008 | | | | | | | | 49 (8) | | | 47 (7) | | | 0.055 | | | 0.012 | |
| arterial diseases**^*^** | 2,455 (17) | | 2,577 (18) | | | | | | | | 0.040 | | | | | | | | 0.022 | | | | | | | | 114 (17) | | | 112 (17) | | | 0.017 | | | 0.008 | |
| heart failure | 6,521 (46) | | 6,485 (46) | | | | | | | | 0.004 | | | | | | | | 0.005 | | | | | | | | 336 (52) | | | 321 (49) | | | 0.04 | | | 0.045 | |
| CKD | 530 (4) | | 616 (4) | | | | | | | | 0.045 | | | | | | | | 0.031 | | | | | | | | 35 (5) | | | 40 (6) | | | 0.057 | | | 0.033 | |
| CHA2DS2-VASc |  | |  | | | | | | | |  | | | | | | | |  | | | | | | | |  | | |  | | |  | | |  | |
| 0-1, n(%) | 1,774 (13) | | 1,915 (14) | | | | | | | | 0.106 | | | | | | | | 0.029 | | | | | | | | 94 (14) | | | 84 (13) | | | 0.036 | | | 0.046 | |
| 2-3, n(%) | 5,339 (38) | | 5,681 (40) | | | | | | | | 0.084 | | | | | | | | 0.050 | | | | | | | | 223 (34) | | | 243 (37) | | | 0.125 | | | 0.063 | |
| ≥4, n(%) | 7,058 (50) | | 6,575 (46) | | | | | | | | 0.155 | | | | | | | | 0.068 | | | | | | | | 335 (51) | | | 325 (50) | | | 0.096 | | | 0.030 | |
| Medications, n(%) |  | |  | | | | | | | |  | | | | | | | |  | | | | | | | |  | | |  | | |  | | |  | |
| antiplatelet agent | 5,347 (38) | | 5,627 (40) | | | | | | | | 0.085 | | | | | | | | 0.041 | | | | | | | | 249 (38) | | | 250 (38) | | | 0.056 | | | 0.003 | |
| statin | 8,446 (60) | | 8,821 (62) | | | | | | | | 0.093 | | | | | | | | 0.054 | | | | | | | | 340 (52) | | | 332 (51) | | | 0.050 | | | 0.025 | |
| ACEI/ARB | 8,053 (57) | | 8,046 (57) | | | | | | | | 0.013 | | | | | | | | 0.001 | | | | | | | | 350 (54) | | | 359 (55) | | | 0.018 | | | 0.028 | |
| beta blocker | 6,929 (49) | | 7,159 (51) | | | | | | | | 0.04 | | | | | | | | 0.032 | | | | | | | | 322 (49) | | | 324 (50) | | | 0.042 | | | 0.006 | |
| CCB | 5,911 (42) | | 5,985 (42) | | | | | | | | 0.029 | | | | | | | | 0.011 | | | | | | | | 258 (40) | | | 264 (40) | | | 0.024 | | | 0.019 | |
| Diuretics | 1,399 (10) | | 1,447 (10) | | | | | | | | 0.021 | | | | | | | | 0.011 | | | | | | | | 80 (12) | | | 82 (13) | | | 0.005 | | | 0.009 | |
| **B. Apixaban vs. Once-daily regimen** | | | | | | | | | | | | | | | | | | | | | | | | | | | | | | | | | | | | | |
| **Characteristics** | **Composite outcome** | | | | | | | | | | | | | | | | | | | | | | | | | | | | | | | | | | | | |
|  | **High adherence group** | | | | | | | | | | | | | | | | | | | | | | | | | | **Low adherence group** | | | | | | | | | | |
|  | Apixaban | | Once-daily | | | | | | | | *d_before_* | | | | | | | | *d_after_* | | | | | | | | Apixaban | | | Once-daily | | | *d_before_* | | | *d_after_* | |
| Subjects, n | 9,612 | | 9,612 | | | | | | | |  | | | | | | | |  | | | | | | | | 400 | | | 400 | | |  | | |  | |
| Age, years | 74 ± 11 | | 73 ± 10 | | | | | | | | 0.151 | | | | | | | | 0.018 | | | | | | | | 73 ± 13 | | | 73 ± 13 | | | 0.019 | | | 0.027 | |
| Male, n(%) | 4,637 (48) | | 4,684 (49) | | | | | | | | 0.108 | | | | | | | | 0.01 | | | | | | | | 211 (53) | | | 206 (52) | | | 0.02 | | | 0.025 | |
| Medical history, n(%) |  | |  | | | | | | | |  | | | | | | | |  | | | | | | | |  | | |  | | |  | | |  | |
| hypertension | 7,752 (81) | | 7,713 (80) | | | | | | | | 0.003 | | | | | | | | 0.01 | | | | | | | | 310 (78) | | | 318 (80) | | | 0.001 | | | 0.048 | |
| diabetes | 2,533 (26) | | 2,476 (26) | | | | | | | | 0.024 | | | | | | | | 0.013 | | | | | | | | 92 (23) | | | 86(22) | | | 0.037 | | | 0.036 | |
| dyslipidemia | 5,872 (61) | | 5,803 (60) | | | | | | | | 0.056 | | | | | | | | 0.015 | | | | | | | | 194 (49) | | | 191 (48) | | | 0.013 | | | 0.015 | |
| myocardial infarction | 1,000 (10) | | 948 (10) | | | | | | | | 0.077 | | | | | | | | 0.019 | | | | | | | | 44 (11) | | | 45 (11) | | | 0.091 | | | 0.008 | |
| stroke | 3,348 (35) | | 3,311 (34) | | | | | | | | 0.175 | | | | | | | | 0.008 | | | | | | | | 119 (30) | | | 124 (31) | | | 0.003 | | | 0.027 | |
| thromboembolism | 455 (5) | | 465 (5) | | | | | | | | 0.025 | | | | | | | | 0.005 | | | | | | | | 35 (9) | | | 32 (8) | | | 0.031 | | | 0.027 | |
| arterial diseases**^*^** | 1,757 (18) | | 1,730 (18) | | | | | | | | 0.029 | | | | | | | | 0.007 | | | | | | | | 70 (18) | | | 73 (18) | | | 0.054 | | | 0.019 | |
| heart failure | 4,573 (48) | | 4,542 (47) | | | | | | | | 0.04 | | | | | | | | 0.006 | | | | | | | | 200 (50) | | | 191 (48) | | | 0.004 | | | 0.045 | |
| CKD | 523 (5) | | 477 (5) | | | | | | | | 0.09 | | | | | | | | 0.023 | | | | | | | | 34 (9) | | | 32 (8) | | | 0.18 | | | 0.019 | |
| CHA2DS2-VASc |  | |  | | | | | | | |  | | | | | | | |  | | | | | | | |  | | |  | | |  | | |  | |
| 0-1, n(%) | 989 (10) | | 1,047 (11) | | | | | | | | 0.151 | | | | | | | | 0.018 | | | | | | | | 51 (13) | | | 51 (13) | | | 0.047 | | | <0.001 | |
| 2-3, n(%) | 3,523 (37) | | 3,515 (37) | | | | | | | | 0.091 | | | | | | | | 0.002 | | | | | | | | 120 (30) | | | 123 (31) | | | 0.162 | | | 0.016 | |
| ≥4, n(%) | 5,100 (53) | | 5,050 (53) | | | | | | | | 0.19 | | | | | | | | 0.01 | | | | | | | | 229 (57) | | | 226 (57) | | | 0.122 | | | 0.015 | |
| Medications, n(%) |  | |  | | | | | | | |  | | | | | | | |  | | | | | | | |  | | |  | | |  | | |  | |
| antiplatelet agent | 3,536 (37) | | 3,565 (37) | | | | | | | | 0.081 | | | | | | | | 0.006 | | | | | | | | 151 (38) | | | 153 (38) | | | 0.02 | | | 0.01 | |
| statin | 6,022 (63) | | 5,936 (62) | | | | | | | | 0.086 | | | | | | | | 0.018 | | | | | | | | 208 (52) | | | 207 (52) | | | 0.045 | | | 0.005 | |
| ACEI/ARB | 5,505 (57) | | 5,431 (57) | | | | | | | | 0.006 | | | | | | | | 0.016 | | | | | | | | 221 (55) | | | 231 (58) | | | 0.014 | | | 0.05 | |
| beta blocker | 4,864 (51) | | 4,918 (51) | | | | | | | | 0.062 | | | | | | | | 0.011 | | | | | | | | 206 (52) | | | 212 (53) | | | 0.063 | | | 0.03 | |
| CCB | 4,217 (44) | | 4,154 (43) | | | | | | | | 0.046 | | | | | | | | 0.013 | | | | | | | | 164 (41) | | | 177 (44) | | | 0.04 | | | 0.066 | |
| Diuretics | 1,105 (11) | | 1,084 (11) | | | | | | | | 0.058 | | | | | | | | 0.007 | | | | | | | | 51 (13) | | | 50 (13) | | | 0.006 | | | 0.008 | |
| **Characteristics** | **Stroke** | | | | | | | | | | | | | | | | | | | | | | | | | | | | | | | | | | | | |
|  | **High adherence group** | | | | | | | | | | | | | | | | | | | | | | | | | | **Low adherence group** | | | | | | | | | | |
|  | Apixaban | | | Once-daily | | | | | | | | *d_before_* | | | | | | | | *d_after_* | | | | | | | Apixaban | | | Once-daily | | | *d_before_* | | | *d_after_* | |
| Subjects, n | 9,608 | | | 9,608 | | | | | | | |  | | | | | | | |  | | | | | | | 397 | | | 397 | | |  | | |  | |
| Age, years | 74 ± 11 | | | 73 ± 10 | | | | | | | | 0.150 | | | | | | | | 0.022 | | | | | | | 73 ± 13 | | | 73 ± 14 | | | 0.016 | | | 0.015 | |
| Male, n(%) | 4,632 (48) | | | 4,703 (49) | | | | | | | | 0.109 | | | | | | | | 0.015 | | | | | | | 211 (53) | | | 219 (55) | | | 0.019 | | | 0.040 | |
| Medical history, n(%) |  | | |  | | | | | | | |  | | | | | | | |  | | | | | | |  | | |  | | |  | | |  | |
| hypertension | 7,747 (81) | | | 7,752 (81) | | | | | | | | 0.002 | | | | | | | | 0.001 | | | | | | | 309 (78) | | | 310 (78) | | | 0.005 | | | 0.006 | |
| diabetes | 2,530 (26) | | | 2,477 (26) | | | | | | | | 0.024 | | | | | | | | 0.013 | | | | | | | 92 (23) | | | 89 (22) | | | 0.04 | | | 0.018 | |
| dyslipidemia | 5,869 (61) | | | 5,819 (61) | | | | | | | | 0.056 | | | | | | | | 0.011 | | | | | | | 192 (48) | | | 194 (49) | | | 0.01 | | | 0.01 | |
| myocardial infarction | 993 (10) | | | 976 (10) | | | | | | | | 0.076 | | | | | | | | 0.006 | | | | | | | 46 (12) | | | 46 (12) | | | 0.108 | | | <0.001 | |
| stroke | 3,347 (35) | | | 3,312 (34) | | | | | | | | 0.175 | | | | | | | | 0.008 | | | | | | | 118 (30) | | | 109 (27) | | | 0.009 | | | 0.05 | |
| thromboembolism | 457 (5) | | | 452 (5) | | | | | | | | 0.024 | | | | | | | | 0.002 | | | | | | | 36 (9) | | | 35 (9) | | | 0.041 | | | 0.009 | |
| arterial diseases**^*^** | 1,758 (18) | | | 1,727 (18) | | | | | | | | 0.03 | | | | | | | | 0.008 | | | | | | | 68 (17) | | | 72 (18) | | | 0.065 | | | 0.026 | |
| heart failure | 4,570 (48) | | | 4,504 (47) | | | | | | | | 0.04 | | | | | | | | 0.014 | | | | | | | 201 (51) | | | 214 (54) | | | 0.022 | | | 0.065 | |
| CKD | 522 (5) | | | 488 (5) | | | | | | | | 0.09 | | | | | | | | 0.017 | | | | | | | 33 (8) | | | 29 (7) | | | 0.189 | | | 0.039 | |
| CHA2DS2-VASc |  | | |  | | | | | | | |  | | | | | | | |  | | | | | | |  | | |  | | |  | | |  | |
| 0-1, n(%) | 990 (10) | | | 1,024 (11) | | | | | | | | 0.15 | | | | | | | | 0.011 | | | | | | | 51 (13) | | | 53 (13) | | | 0.01 | | | 0.015 | |
| 2-3, n(%) | 3,523 (37) | | | 3,482 (36) | | | | | | | | 0.091 | | | | | | | | 0.009 | | | | | | | 118 (30) | | | 112 (28) | | | 0.174 | | | 0.032 | |
| ≥4, n(%) | 5,095 (53) | | | 5,102 (53) | | | | | | | | 0.19 | | | | | | | | 0.001 | | | | | | | 228 (57) | | | 232 (58) | | | 0.136 | | | 0.02 | |
| Medications, n(%) |  | | |  | | | | | | | |  | | | | | | | |  | | | | | | |  | | |  | | |  | | |  | |
| antiplatelet agent | 3,538 (37) | | | 3,594 (37) | | | | | | | | 0.08 | | | | | | | | 0.012 | | | | | | | 150 (38) | | | 156 (39) | | | 0.028 | | | 0.031 | |
| statin | 6,017 (63) | | | 5,963 (62) | | | | | | | | 0.085 | | | | | | | | 0.012 | | | | | | | 206 (52) | | | 209 (53) | | | 0.048 | | | 0.015 | |
| ACEI/ARB | 5,504 (57) | | | 5,487 (57) | | | | | | | | 0.005 | | | | | | | | 0.004 | | | | | | | 221 (56) | | | 217 (55) | | | 0.011 | | | 0.02 | |
| beta blocker | 4,857 (51) | | | 4,869 (51) | | | | | | | | 0.061 | | | | | | | | 0.003 | | | | | | | 208 (52) | | | 199 (50) | | | 0.07 | | | 0.045 | |
| CCB | 4,215 (44) | | | 4,150 (43) | | | | | | | | 0.046 | | | | | | | | 0.014 | | | | | | | 164 (41) | | | 166 (42) | | | 0.037 | | | 0.01 | |
| Diuretics | 1,106 (12) | | | 1,068 (11) | | | | | | | | 0.059 | | | | | | | | 0.013 | | | | | | | 51 (13) | | | 49 (12) | | | 0.001 | | | 0.015 | |
| **Characteristics** | **AMI** | | | | | | | | | | | | | | | | | | | | | | | | | | | | | | | | | | | | |
|  | **High adherence group** | | | | | | | | | | | | | | | | | | | | | | | | | | **Low adherence group** | | | | | | | | | | |
|  | Apixaban | | | Once-daily | | | | | | | | *d_before_* | | | | | | | | *d_after_* | | | | | | | Apixaban | | | Once-daily | | | *d_before_* | | | *d_after_* | |
| Subjects, n | 9,579 | | | 9,579 | | | | | | | |  | | | | | | | |  | | | | | | | 392 | | | 392 | | |  | | |  | |
| Age, years | 74 ± 11 | | | 73 ± 10 | | | | | | | | 0.149 | | | | | | | | 0.023 | | | | | | | 73 ± 13 | | | 73 ± 14 | | | 0.014 | | | 0.006 | |
| Male, n(%) | 4,622 (48) | | | 4,682 (49) | | | | | | | | 0.107 | | | | | | | | 0.013 | | | | | | | 213 (54) | | | 214 (55) | | | 0.024 | | | 0.005 | |
| Medical history, n(%) |  | | |  | | | | | | | |  | | | | | | | |  | | | | | | |  | | |  | | |  | | |  | |
| hypertension | 7,720 (81) | | | 7,707 (80) | | | | | | | | 0.003 | | | | | | | | 0.003 | | | | | | | 301 (77) | | | 304 (78) | | | <0.001 | | | 0.018 | |
| diabetes | 2,512 (26) | | | 2,424 (25) | | | | | | | | 0.024 | | | | | | | | 0.021 | | | | | | | 95 (24) | | | 102 (26) | | | 0.045 | | | 0.042 | |
| dyslipidemia | 5,853 (61) | | | 5,798 (60) | | | | | | | | 0.06 | | | | | | | | 0.012 | | | | | | | 189 (48) | | | 178 (45) | | | 0.016 | | | 0.056 | |
| myocardial infarction | 986 (11) | | | 969 (10) | | | | | | | | 0.076 | | | | | | | | 0.006 | | | | | | | 44 (11) | | | 45 (11) | | | 0.077 | | | 0.008 | |
| stroke | 3,302 (34) | | | 3,269 (34) | | | | | | | | 0.175 | | | | | | | | 0.008 | | | | | | | 111 (28) | | | 107 (27) | | | 0.006 | | | 0.022 | |
| thromboembolism | 455 (5) | | | 430 (4) | | | | | | | | 0.025 | | | | | | | | 0.012 | | | | | | | 34 (9) | | | 31 (8) | | | 0.006 | | | 0.027 | |
| arterial diseases**^*^** | 1,752 (18) | | | 1,708 (18) | | | | | | | | 0.029 | | | | | | | | 0.012 | | | | | | | 68 (17) | | | 69 (18) | | | 0.025 | | | 0.007 | |
| heart failure | 4,549 (47) | | | 4,464 (47) | | | | | | | | 0.041 | | | | | | | | 0.018 | | | | | | | 198 (51) | | | 199 (51) | | | 0.023 | | | 0.005 | |
| CKD | 517 (5) | | | 471 (5) | | | | | | | | 0.09 | | | | | | | | 0.023 | | | | | | | 33 (8) | | | 35 (9) | | | 0.163 | | | 0.020 | |
| CHA2DS2-VASc |  | | |  | | | | | | | |  | | | | | | | |  | | | | | | |  | | |  | | |  | | |  | |
| 0-1, n(%) | 993 (10) | | | 1,053 (11) | | | | | | | | 0.149 | | | | | | | | 0.019 | | | | | | | 51 (13) | | | 56 (14) | | | 0.047 | | | 0.038 | |
| 2-3, n(%) | 3,511 (37) | | | 3,459 (36) | | | | | | | | 0.09 | | | | | | | | 0.011 | | | | | | | 120 (31) | | | 120 (31) | | | 0.173 | | | <0.001 | |
| ≥4, n(%) | 5,075 (53) | | | 5,067 (53) | | | | | | | | 0.188 | | | | | | | | 0.002 | | | | | | | 221 (56) | | | 216 (55) | | | 0.133 | | | 0.026 | |
| Medications, n(%) |  | | |  | | | | | | | |  | | | | | | | |  | | | | | | |  | | |  | | |  | | |  | |
| antiplatelet agent | 3,533 (37) | | | 3,594 (38) | | | | | | | | 0.082 | | | | | | | | 0.013 | | | | | | | 150 (38) | | | 158 (40) | | | 0.002 | | | 0.042 | |
| statin | 5,996 (63) | | | 5,932 (62) | | | | | | | | 0.089 | | | | | | | | 0.014 | | | | | | | 203 (52) | | | 193 (49) | | | 0.024 | | | 0.051 | |
| ACEI/ARB | 5,476 (57) | | | 5,370 (56) | | | | | | | | 0.007 | | | | | | | | 0.022 | | | | | | | 213 (54) | | | 221 (56) | | | 0.013 | | | 0.041 | |
| beta blocker | 4,843 (51) | | | 4,887 (51) | | | | | | | | 0.061 | | | | | | | | 0.009 | | | | | | | 204 (52) | | | 204 (52) | | | 0.086 | | | <0.001 | |
| CCB | 4,203 (44) | | | 4,159 (43) | | | | | | | | 0.047 | | | | | | | | 0.009 | | | | | | | 156 (40) | | | 156 (40) | | | 0.002 | | | <0.001 | |
| Diuretics | 1,089 (11) | | | 1,067 (11) | | | | | | | | 0.055 | | | | | | | | 0.007 | | | | | | | 51 (13) | | | 46 (12) | | | 0.003 | | | 0.038 | |
| **Characteristics** | **Death** | | | | | | | | | | | | | | | | | | | | | | | | | | | | | | | | | | | | |
|  | **High adherence group** | | | | | | | | | | | | | | | | | | | | | | | | | | | **Low adherence group** | | | | | | | | | |
|  | Apixaban | | | | Once-daily | | | | | | | | *d_before_* | | | | | | | | *d_after_* | | | | | | | Apixaban | | | Once-daily | | | *d_before_* | | | *d_after_* |
| Subjects, n | 9,572 | | | | 9,572 | | | | | | | |  | | | | | | | |  | | | | | | | 389 | | | 389 | | |  | | |  |
| Age, years | 73 ± 11 | | | | 73 ± 10 | | | | | | | | 0.148 | | | | | | | | 0.016 | | | | | | | 73 ± 13 | | | 72 ± 14 | | | 0.01 | | | 0.037 |
| Male, n(%) | 4,614 (48) | | | | 4,672 (49) | | | | | | | | 0.108 | | | | | | | | 0.012 | | | | | | | 213 (55) | | | 210 (54) | | | 0.024 | | | 0.015 |
| Medical history, n(%) |  | | | |  | | | | | | | |  | | | | | | | |  | | | | | | |  | | |  | | |  | | |  |
| hypertension | 7,712 (81) | | | | 7,690 (80) | | | | | | | | 0.002 | | | | | | | | 0.006 | | | | | | | 299 (77) | | | 302 (78) | | | 0.006 | | | 0.018 |
| diabetes | 2,508 (26) | | | | 2,431 (25) | | | | | | | | 0.023 | | | | | | | | 0.018 | | | | | | | 94 (24) | | | 93 (24) | | | 0.048 | | | 0.006 |
| dyslipidemia | 5,848 (61) | | | | 5,790 (60) | | | | | | | | 0.060 | | | | | | | | 0.012 | | | | | | | 187 (48) | | | 188 (48) | | | 0.014 | | | 0.005 |
| myocardial infarction | 977 (10) | | | | 939 (10) | | | | | | | | 0.074 | | | | | | | | 0.014 | | | | | | | 44 (11) | | | 48 (12) | | | 0.094 | | | 0.034 |
| stroke | 3,300 (34) | | | | 3,285 (34) | | | | | | | | 0.175 | | | | | | | | 0.003 | | | | | | | 109 (28) | | | 120 (31) | | | <0.001 | | | 0.063 |
| thromboembolism | 457 (5) | | | | 455 (5) | | | | | | | | 0.025 | | | | | | | | <0.001 | | | | | | | 34 (9) | | | 36 (9) | | | 0.016 | | | 0.019 |
| arterial diseases**^*^** | 1,751 (18) | | | | 1,711 (18) | | | | | | | | 0.029 | | | | | | | | 0.011 | | | | | | | 67 (17) | | | 68 (17) | | | 0.035 | | | 0.007 |
| heart failure | 4,543 (47) | | | | 4,520 (47) | | | | | | | | 0.040 | | | | | | | | 0.005 | | | | | | | 197 (51) | | | 189 (49) | | | 0.042 | | | 0.041 |
| CKD | 515 (5) | | | | 477 (5) | | | | | | | | 0.089 | | | | | | | | 0.019 | | | | | | | 33 (8) | | | 30 (8) | | | 0.172 | | | 0.030 |
| CHA2DS2-VASc |  | | | |  | | | | | | | |  | | | | | | | |  | | | | | | |  | | |  | | |  | | |  |
| 0-1, n(%) | 993 (10) | | | | 1,074 (11) | | | | | | | | 0.149 | | | | | | | | 0.025 | | | | | | | 52 (13) | | | 56 (14) | | | 0.042 | | | 0.031 |
| 2-3, n(%) | 3,511 (37) | | | | 3,448 (36) | | | | | | | | 0.089 | | | | | | | | 0.014 | | | | | | | 118 (30) | | | 120 (31) | | | 0.185 | | | 0.011 |
| ≥4, n(%) | 5,068 (53) | | | | 5,050 (53) | | | | | | | | 0.187 | | | | | | | | 0.004 | | | | | | | 219 (56) | | | 213 (55) | | | 0.147 | | | 0.031 |
| Medications, n(%) |  | | | |  | | | | | | | |  | | | | | | | |  | | | | | | |  | | |  | | |  | | |  |
| antiplatelet agent | 3,535 (37) | | | | 3,608 (38) | | | | | | | | 0.082 | | | | | | | | 0.016 | | | | | | | 150 (39) | | | 144 (37) | | | 0.010 | | | 0.030 |
| statin | 5,989 (63) | | | | 5,912 (62) | | | | | | | | 0.089 | | | | | | | | 0.016 | | | | | | | 201 (52) | | | 203 (52) | | | 0.027 | | | 0.010 |
| ACEI/ARB | 5,473 (57) | | | | 5,384 (56) | | | | | | | | 0.006 | | | | | | | | 0.019 | | | | | | | 210 (54) | | | 207 (53) | | | 0.01 | | | 0.015 |
| beta blocker | 4,834 (51) | | | | 4,877 (51) | | | | | | | | 0.060 | | | | | | | | 0.009 | | | | | | | 203 (52) | | | 209 (54) | | | 0.09 | | | 0.031 |
| CCB | 4,201 (44) | | | | 4,145 (43) | | | | | | | | 0.047 | | | | | | | | 0.012 | | | | | | | 152 (39) | | | 164 (42) | | | <0.001 | | | 0.063 |
| Diuretics | 1,089 (11) | | | | 1,061 (11) | | | | | | | | 0.055 | | | | | | | | 0.010 | | | | | | | 51 (13) | | | 44 (11) | | | 0.004 | | | 0.053 |
| **C. Dabigatran vs. Once-daily regimen** | | | | | | | | | | | | | | | | | | | | | | | | | | | | | | | | | | | | | |
| **Characteristics** | **Composite outcome** | | | | | | | | | | | | | | | | | | | | | | | | | | | | | | | | | | | | |
|  | **High adherence group** | | | | | | | | | | | | | | | | | | | | | | | | | | | **Low adherence group** | | | | | | | | | |
|  | Dabigatran | | | | Once-daily | | | | | | | | *d_before_* | | | | | | | | *d_after_* | | | | | | | Dabigatran | | | Once-daily | | | *d_before_* | | | *d_after_* |
| Subjects, n | 6,853 | | | | 6,853 | | | | | | | |  | | | | | | | |  | | | | | | | 449 | | | 449 | | |  | | |  |
| Age, years | 71 ± 11 | | | | 71 ± 11 | | | | | | | | 0.06 | | | | | | | | 0.014 | | | | | | | 71 ± 13 | | | 72 ± 14 | | | 0.153 | | | 0.063 |
| Male, n(%) | 3,857 (56) | | | | 3,880 (57) | | | | | | | | 0.053 | | | | | | | | 0.007 | | | | | | | 232 (52) | | | 229 (51) | | | 0.007 | | | 0.013 |
| Medical history, n(%) |  | | | |  | | | | | | | |  | | | | | | | |  | | | | | | |  | | |  | | |  | | |  |
| hypertension | 5,455 (80) | | | | 5,447 (79) | | | | | | | | 0.024 | | | | | | | | 0.003 | | | | | | | 341 (76) | | | 338 (75) | | | 0.048 | | | 0.016 |
| diabetes | 1,738 (25) | | | | 1,722 (25) | | | | | | | | 0.001 | | | | | | | | 0.005 | | | | | | | 101 (22) | | | 92 (20) | | | 0.014 | | | 0.048 |
| dyslipidemia | 4,282 (62) | | | | 4,246 (62) | | | | | | | | 0.085 | | | | | | | | 0.011 | | | | | | | 226 (50) | | | 209 (47) | | | 0.038 | | | 0.076 |
| myocardial infarction | 522 (8) | | | | 532 (8) | | | | | | | | 0.02 | | | | | | | | 0.005 | | | | | | | 42 (9) | | | 36 (8) | | | 0.006 | | | 0.046 |
| stroke | 2,474 (36) | | | | 2,439 (36) | | | | | | | | 0.201 | | | | | | | | 0.011 | | | | | | | 128 (29) | | | 126 (28) | | | 0.029 | | | 0.01 |
| thromboembolism | 361 (5) | | | | 352 (5) | | | | | | | | <0.001 | | | | | | | | 0.006 | | | | | | | 21 (5) | | | 23 (5) | | | 0.148 | | | 0.018 |
| arterial diseases**^*^** | 1,318 (19) | | | | 1,229 (18) | | | | | | | | 0.054 | | | | | | | | 0.034 | | | | | | | 95 (21) | | | 83 (18) | | | 0.049 | | | 0.066 |
| heart failure | 2,971 (43) | | | | 2,974 (43) | | | | | | | | 0.045 | | | | | | | | <0.001 | | | | | | | 226 (50) | | | 227 (51) | | | 0.01 | | | 0.004 |
| CKD | 209 (3) | | | | 197 (3) | | | | | | | | 0.03 | | | | | | | | 0.01 | | | | | | | 15 (3) | | | 15 (3) | | | 0.082 | | | <0.001 |
| CHA2DS2-VASc |  | | | |  | | | | | | | |  | | | | | | | |  | | | | | | |  | | |  | | |  | | |  |
| 0-1, n(%) | 922 (13) | | | | 951 (14) | | | | | | | | 0.053 | | | | | | | | 0.012 | | | | | | | 59 (13) | | | 56 (12) | | | 0.051 | | | 0.02 |
| 2-3, n(%) | 2,548 (37) | | | | 2,552 (37) | | | | | | | | 0.081 | | | | | | | | 0.001 | | | | | | | 158 (35) | | | 161 (36) | | | 0.038 | | | 0.014 |
| ≥4, n(%) | 3,383 (49) | | | | 3,350 (49) | | | | | | | | 0.116 | | | | | | | | 0.01 | | | | | | | 232 (52) | | | 232 (52) | | | 0.003 | | | <0.001 |
| Medications, n(%) |  | | | |  | | | | | | | |  | | | | | | | |  | | | | | | |  | | |  | | |  | | |  |
| antiplatelet agent | 2,498 (36) | | | | 2,470 (36) | | | | | | | | 0.088 | | | | | | | | 0.008 | | | | | | | 155 (35) | | | 156 (35) | | | 0.081 | | | 0.005 |
| statin | 4,329 (63) | | | | 4,285 (63) | | | | | | | | 0.097 | | | | | | | | 0.013 | | | | | | | 238 (53) | | | 222 (49) | | | 0.055 | | | 0.071 |
| ACEI/ARB | 3,872 (57) | | | | 3,860 (56) | | | | | | | | 0.021 | | | | | | | | 0.004 | | | | | | | 240 (53) | | | 233 (52) | | | 0.059 | | | 0.031 |
| beta blocker | 3,289 (48) | | | | 3,305 (48) | | | | | | | | 0.009 | | | | | | | | 0.005 | | | | | | | 222 (49) | | | 228 (51) | | | 0.008 | | | 0.027 |
| CCB | 2,860 (42) | | | | 2,888 (42) | | | | | | | | 0.003 | | | | | | | | 0.008 | | | | | | | 185 (41) | | | 181 (40) | | | 0.033 | | | 0.018 |
| Diuretics | 613 (9) | | | | 603 (9) | | | | | | | | 0.026 | | | | | | | | 0.005 | | | | | | | 54 (12) | | | 51 (11) | | | 0.02 | | | 0.020 |
| **Characteristics** | **Stroke** | | | | | | | | | | | | | | | | | | | | | | | | | | | | | | | | | | | | |
|  | **High adherence group** | | | | | | | | | | | | | | | | | | | | | | | | | | | **Low adherence group** | | | | | | | | | |
|  | Dabigatran | | | | | Once-daily | | | | | | | | *d_before_* | | | | | | | | *d_after_* | | | | | | Dabigatran | | | Once-daily | | | *d_before_* | | | *d_after_* |
| Subjects, n | 6,850 | | | | | 6,850 | | | | | | | |  | | | | | | | |  | | | | | | 449 | | | 449 | | |  | | |  |
| Age, years | 71 ± 11 | | | | | 71 ± 11 | | | | | | | | 0.059 | | | | | | | | 0.002 | | | | | | 71 ± 13 | | | 71 ± 15 | | | 0.152 | | | 0.001 |
| Male, n(%) | 3,855 (56) | | | | | 3,867 (56) | | | | | | | | 0.053 | | | | | | | | 0.004 | | | | | | 234 (52) | | | 234 (52) | | | 0.004 | | | <0.001 |
| Medical history, n(%) |  | | | | |  | | | | | | | |  | | | | | | | |  | | | | | |  | | |  | | |  | | |  |
| hypertension | 5,451 (80) | | | | | 5,442 (79) | | | | | | | | 0.024 | | | | | | | | 0.003 | | | | | | 341 (76) | | | 343 (76) | | | 0.047 | | | 0.011 |
| diabetes | 1,734 (25) | | | | | 1,694 (25) | | | | | | | | <0.001 | | | | | | | | 0.013 | | | | | | 101 (22) | | | 89 (20) | | | 0.015 | | | 0.064 |
| dyslipidemia | 4,279 (62) | | | | | 4,292 (63) | | | | | | | | 0.085 | | | | | | | | 0.004 | | | | | | 227 (51) | | | 215 (48) | | | 0.043 | | | 0.053 |
| myocardial infarction | 520 (8) | | | | | 510 (7) | | | | | | | | 0.02 | | | | | | | | 0.005 | | | | | | 42 (9) | | | 38 (8) | | | 0.015 | | | 0.031 |
| stroke | 2,473 (36) | | | | | 2,430 (35) | | | | | | | | 0.201 | | | | | | | | 0.014 | | | | | | 128 (29) | | | 119 (27) | | | 0.03 | | | 0.044 |
| thromboembolism | 360 (5) | | | | | 328 (5) | | | | | | | | 0.001 | | | | | | | | 0.021 | | | | | | 21 (5) | | | 21 (5) | | | 0.139 | | | <0.001 |
| arterial diseases**^*^** | 1,320 (19) | | | | | 1,293 (19) | | | | | | | | 0.055 | | | | | | | | 0.01 | | | | | | 94 (21) | | | 89 (20) | | | 0.039 | | | 0.028 |
| heart failure | 2,969 (43) | | | | | 2,967 (43) | | | | | | | | 0.045 | | | | | | | | <0.001 | | | | | | 225 (50) | | | 228 (51) | | | 0.016 | | | 0.013 |
| CKD | 210 (3) | | | | | 205 (3) | | | | | | | | 0.029 | | | | | | | | 0.004 | | | | | | 15 (3) | | | 16 (4) | | | 0.083 | | | 0.011 |
| CHA2DS2-VASc |  | | | | |  | | | | | | | |  | | | | | | | |  | | | | | |  | | |  | | |  | | |  |
| 0-1, n(%) | 921 (13) | | | | | 986 (14) | | | | | | | | 0.053 | | | | | | | | 0.027 | | | | | | 59 (13) | | | 55 (12) | | | 0.045 | | | 0.027 |
| 2-3, n(%) | 2,548 (37) | | | | | 2,532 (37) | | | | | | | | 0.08 | | | | | | | | 0.005 | | | | | | 158 (35) | | | 160 (36) | | | 0.04 | | | 0.009 |
| ≥4, n(%) | 3,381 (49) | | | | | 3,332 (49) | | | | | | | | 0.116 | | | | | | | | 0.014 | | | | | | 232 (52) | | | 234 (52) | | | 0.009 | | | 0.009 |
| Medications, n(%) |  | | | | |  | | | | | | | |  | | | | | | | |  | | | | | |  | | |  | | |  | | |  |
| antiplatelet agent | 2,498 (36) | | | | | 2,540 (37) | | | | | | | | 0.088 | | | | | | | | 0.013 | | | | | | 155 (35) | | | 155 (35) | | | 0.091 | | | <0.001 |
| statin | 4,326 (63) | | | | | 4,348 (63) | | | | | | | | 0.096 | | | | | | | | 0.007 | | | | | | 238 (53) | | | 227 (51) | | | 0.06 | | | 0.049 |
| ACEI/ARB | 3,867 (56) | | | | | 3,885 (57) | | | | | | | | 0.022 | | | | | | | | 0.005 | | | | | | 241 (54) | | | 241 (54) | | | 0.055 | | | <0.001 |
| beta blocker | 3,287 (48) | | | | | 3,323 (49) | | | | | | | | 0.009 | | | | | | | | 0.011 | | | | | | 220 (49) | | | 225 (50) | | | 0.004 | | | 0.022 |
| CCB | 2,858 (42) | | | | | 2,881 (42) | | | | | | | | 0.003 | | | | | | | | 0.007 | | | | | | 186 (41) | | | 179 (40) | | | 0.029 | | | 0.032 |
| Diuretics | 613 (9) | | | | | 590 (9) | | | | | | | | 0.026 | | | | | | | | 0.012 | | | | | | 55 (12) | | | 48 (11) | | | 0.021 | | | 0.047 |
| **Characteristics** | **AMI** | | | | | | | | | | | | | | | | | | | | | | | | | | | | | | | | | | | | |
|  | **High adherence group** | | | | | | | | | | | | | | | | | | | | | | | | | | | **Low adherence group** | | | | | | | | | |
|  | Dabigatran | | | | | Once-daily | | | | | | | | *d_before_* | | | | | | | | *d_after_* | | | | | | Dabigatran | | | Once-daily | | | *d_before_* | | | *d_after_* |
| Subjects, n | 6,865 | | | | | 6,865 | | | | | | | |  | | | | | | | |  | | | | | | 426 | | | 426 | | |  | | |  |
| Age, years | 71 ± 11 | | | | | 71 ± 11 | | | | | | | | 0.061 | | | | | | | | 0.025 | | | | | | 72 ± 13 | | | 72 ± 14 | | | 0.133 | | | 0.004 |
| Male, n(%) | 3,868 (56) | | | | | 3,890 (57) | | | | | | | | 0.055 | | | | | | | | 0.006 | | | | | | 221 (52) | | | 219 (51) | | | 0.016 | | | 0.009 |
| Medical history, n(%) |  | | | | |  | | | | | | | |  | | | | | | | |  | | | | | |  | | |  | | |  | | |  |
| hypertension | 5,457 (79) | | | | | 5,446 (79) | | | | | | | | 0.025 | | | | | | | | 0.004 | | | | | | 322 (76) | | | 330 (77) | | | 0.029 | | | 0.044 |
| diabetes | 1,732 (25) | | | | | 1,698 (25) | | | | | | | | 0.001 | | | | | | | | 0.011 | | | | | | 94 (22) | | | 91 (21) | | | 0.016 | | | 0.017 |
| dyslipidemia | 4,278 (62) | | | | | 4,250 (62) | | | | | | | | 0.085 | | | | | | | | 0.008 | | | | | | 221 (52) | | | 211 (50) | | | 0.056 | | | 0.047 |
| myocardial infarction | 513 (7) | | | | | 532 (8) | | | | | | | | 0.024 | | | | | | | | 0.01 | | | | | | 42 (10) | | | 39 (9) | | | 0.026 | | | 0.024 |
| stroke | 2,450 (36) | | | | | 2,377 (35) | | | | | | | | 0.201 | | | | | | | | 0.023 | | | | | | 120 (28) | | | 118 (28) | | | 0.011 | | | 0.01 |
| thromboembolism | 362 (5) | | | | | 357 (5) | | | | | | | | 0.001 | | | | | | | | 0.003 | | | | | | 21 (5) | | | 22 (5) | | | 0.136 | | | 0.009 |
| arterial diseases**^*^** | 1,323 (19) | | | | | 1,287 (19) | | | | | | | | 0.054 | | | | | | | | 0.014 | | | | | | 87 (20) | | | 80 (19) | | | 0.073 | | | 0.041 |
| heart failure | 2,963 (43) | | | | | 2,910 (42) | | | | | | | | 0.046 | | | | | | | | 0.016 | | | | | | 219 (51) | | | 217 (51) | | | 0.032 | | | 0.009 |
| CKD | 210 (3) | | | | | 207 (3) | | | | | | | | 0.027 | | | | | | | | 0.002 | | | | | | 16 (4) | | | 12 (3) | | | 0.072 | | | 0.046 |
| CHA2DS2-VASc |  | | | | |  | | | | | | | |  | | | | | | | |  | | | | | |  | | |  | | |  | | |  |
| 0-1, n(%) | 932 (14) | | | | | 997 (15) | | | | | | | | 0.05 | | | | | | | | 0.027 | | | | | | 53 (12) | | | 50 (12) | | | 0.036 | | | 0.021 |
| 2-3, n(%) | 2,556 (37) | | | | | 2,544 (37) | | | | | | | | 0.078 | | | | | | | | 0.004 | | | | | | 151 (35) | | | 160 (38) | | | 0.069 | | | 0.044 |
| ≥4, n(%) | 3,377 (49) | | | | | 3,324 (48) | | | | | | | | 0.112 | | | | | | | | 0.016 | | | | | | 222 (52) | | | 216 (51) | | | 0.043 | | | 0.028 |
| Medications, n(%) |  | | | | |  | | | | | | | |  | | | | | | | |  | | | | | |  | | |  | | |  | | |  |
| antiplatelet agent | 2,507 (37) | | | | | 2,484 (36) | | | | | | | | 0.090 | | | | | | | | 0.007 | | | | | | 146 (34) | | | 147 (35) | | | 0.087 | | | 0.005 |
| statin | 4,327 (63) | | | | | 4,296 (63) | | | | | | | | 0.098 | | | | | | | | 0.009 | | | | | | 232 (54) | | | 224 (53) | | | 0.066 | | | 0.038 |
| ACEI/ARB | 3,871 (56) | | | | | 3,881 (57) | | | | | | | | 0.022 | | | | | | | | 0.003 | | | | | | 225 (53) | | | 232 (54) | | | 0.031 | | | 0.033 |
| beta blocker | 3,302 (48) | | | | | 3,288 (48) | | | | | | | | 0.012 | | | | | | | | 0.004 | | | | | | 208 (49) | | | 214 (50) | | | <0.001 | | | 0.028 |
| CCB | 2,862 (42) | | | | | 2,867 (42) | | | | | | | | 0.003 | | | | | | | | 0.001 | | | | | | 175 (41) | | | 190 (45) | | | 0.050 | | | 0.072 |
| Diuretics | 608 (9) | | | | | 608 (9) | | | | | | | | 0.028 | | | | | | | | <0.001 | | | | | | 54 (13) | | | 51 (12) | | | 0.012 | | | 0.021 |
| **Characteristics** | **Death** | | | | | | | | | | | | | | | | | | | | | | | | | | | | | | | | | | | | |
|  | **High adherence group** | | | | | | | | | | | | | | | | | | | | | | | | | | | **Low adherence group** | | | | | | | | | |
|  | Dabigatran | | | | | | Once-daily | | | | | | | | *d_before_* | | | | | | | | *d_after_* | | | | | Dabigatran | | | Once-daily | | | *d_before_* | | | *d_after_* |
| Subjects, n | 6,860 | | | | | | 6,860 | | | | | | | |  | | | | | | | |  | | | | | 425 | | | 425 | | |  | | |  |
| Age, years | 71 ± 11 | | | | | | 71 ± 11 | | | | | | | | 0.061 | | | | | | | | 0.014 | | | | | 72 ± 13 | | | 72 ± 15 | | | 0.131 | | | 0.008 |
| Male, n(%) | 3,866 (56) | | | | | | 3,873 (56) | | | | | | | | 0.056 | | | | | | | | 0.002 | | | | | 223 (52) | | | 218 (51) | | | 0.020 | | | 0.024 |
| Medical history, n(%) |  | | | | | |  | | | | | | | |  | | | | | | | |  | | | | |  | | |  | | |  | | |  |
| hypertension | 5,452 (79) | | | | | | 5,450 (79) | | | | | | | | 0.026 | | | | | | | | <0.001 | | | | | 322 (76) | | | 320 (75) | | | 0.028 | | | 0.011 |
| diabetes | 1,727 (25) | | | | | | 1,735 (25) | | | | | | | | <0.001 | | | | | | | | 0.003 | | | | | 96 (23) | | | 95 (22) | | | 0.014 | | | 0.006 |
| dyslipidemia | 4,273 (62) | | | | | | 4,282 (62) | | | | | | | | 0.085 | | | | | | | | 0.003 | | | | | 222 (52) | | | 214 (50) | | | 0.061 | | | 0.038 |
| myocardial infarction | 509 (7) | | | | | | 495 (7) | | | | | | | | 0.025 | | | | | | | | 0.008 | | | | | 43 (10) | | | 35 (8) | | | 0.035 | | | 0.064 |
| stroke | 2,447 (36) | | | | | | 2,394 (35) | | | | | | | | 0.200 | | | | | | | | 0.017 | | | | | 119 (28) | | | 126 (30) | | | 0.012 | | | 0.037 |
| thromboembolism | 361 (5) | | | | | | 347 (5) | | | | | | | | 0.002 | | | | | | | | 0.009 | | | | | 21 (5) | | | 23 (5) | | | 0.126 | | | 0.019 |
| arterial diseases**^*^** | 1,325 (19) | | | | | | 1,264 (18) | | | | | | | | 0.055 | | | | | | | | 0.023 | | | | | 86 (20) | | | 87 (20) | | | 0.062 | | | 0.006 |
| heart failure | 2,960 (43) | | | | | | 2,944 (43) | | | | | | | | 0.046 | | | | | | | | 0.005 | | | | | 218 (51) | | | 211 (50) | | | 0.038 | | | 0.033 |
| CKD | 211 (3) | | | | | | 204 (3) | | | | | | | | 0.026 | | | | | | | | 0.006 | | | | | 16 (4) | | | 13(3) | | | 0.073 | | | 0.034 |
| CHA2DS2-VASc |  | | | | | |  | | | | | | | |  | | | | | | | |  | | | | |  | | |  | | |  | | |  |
| 0-1, n(%) | 931 (14) | | | | | | 978 (14) | | | | | | | | 0.050 | | | | | | | | 0.019 | | | | | 55 (13) | | | 58 (14) | | | 0.030 | | | 0.029 |
| 2-3, n(%) | 2,556 (37) | | | | | | 2,589 (38) | | | | | | | | 0.077 | | | | | | | | 0.010 | | | | | 146 (34) | | | 147 (35) | | | 0.072 | | | 0.005 |
| ≥4, n(%) | 3,373 (49) | | | | | | 3,293 (48) | | | | | | | | 0.111 | | | | | | | | 0.023 | | | | | 225 (53) | | | 220 (52) | | | 0.049 | | | 0.024 |
| Medications, n(%) |  | | | | | |  | | | | | | | |  | | | | | | | |  | | | | |  | | |  | | |  | | |  |
| antiplatelet agent | 2,507(37) | | | | | | 2,536 (37) | | | | | | | | 0.090 | | | | | | | | 0.009 | | | | | 143 (34) | | | 151 (36) | | | 0.098 | | | 0.040 |
| statin | 4,322 (63) | | | | | | 4,326 (63) | | | | | | | | 0.098 | | | | | | | | 0.001 | | | | | 233 (55) | | | 225 (53) | | | 0.071 | | | 0.038 |
| ACEI/ARB | 3,865 (56) | | | | | | 3,919 (57) | | | | | | | | 0.023 | | | | | | | | 0.016 | | | | | 228 (54) | | | 230 (54) | | | 0.026 | | | 0.009 |
| beta blocker | 3,297 (48) | | | | | | 3,329 (49) | | | | | | | | 0.011 | | | | | | | | 0.009 | | | | | 207 (49) | | | 212 (50) | | | 0.005 | | | 0.024 |
| CCB | 2,859 (42) | | | | | | 2,876 (42) | | | | | | | | 0.003 | | | | | | | | 0.005 | | | | | 175 (41) | | | 172 (40) | | | 0.046 | | | 0.014 |
| Diuretics | 607 (9) | | | | | | 564 (8) | | | | | | | | 0.029 | | | | | | | | 0.022 | | | | | 54 (13) | | | 56 (13) | | | 0.014 | | | 0.014 |
| **D. Dabigatran vs. Apixaban** | | | | | | | | | | | | | | | | | | | | | | | | | | | | | | | | | | | | | |
| **Characteristics** | **Composite outcome** | | | | | | | | | | | | | | | | | | | | | | | | | | | | | | | | | | | | |
|  | **High adherence group** | | | | | | | | | | | | | | | | | | | | | | | | | | | **Low adherence group** | | | | | | | | | |
|  | Dabigatran | | | | | | Apixaban | | | | | | | | *d_before_* | | | | | | | | *d_after_* | | | | | Dabigatran | | | Apixaban | | | *d_before_* | | | *d_after_* |
| Subjects, n | 6,779 | | | | | | 6,779 | | | | | | | |  | | | | | | | |  | | | | | 379 | | | 379 | | |  | | |  |
| Age, years | 71 ± 11 | | | | | | 72 ± 11 | | | | | | | | 0.210 | | | | | | | | 0.062 | | | | | 71 ± 13 | | | 72 ± 13 | | | 0.135 | | | 0.074 |
| Male, n(%) | 3,786 (56) | | | | | | 3,646 (54) | | | | | | | | 0.162 | | | | | | | | 0.041 | | | | | 200 (53) | | | 202 (53) | | | 0.013 | | | 0.011 |
| Medical history, n(%) |  | | | | | |  | | | | | | | |  | | | | | | | |  | | | | |  | | |  | | |  | | |  |
| hypertension | 5,397 (80) | | | | | | 5,427 (80) | | | | | | | | 0.027 | | | | | | | | 0.011 | | | | | 282 (74) | | | 294 (78) | | | 0.047 | | | 0.075 |
| diabetes | 1,724 (25) | | | | | | 1,750 (26) | | | | | | | | 0.023 | | | | | | | | 0.009 | | | | | 83 (22) | | | 87 (23) | | | 0.024 | | | 0.025 |
| dyslipidemia | 4,234 (62) | | | | | | 4,239 (63) | | | | | | | | 0.029 | | | | | | | | 0.002 | | | | | 183 (48) | | | 187 (49) | | | 0.051 | | | 0.021 |
| myocardial infarction | 521 (8) | | | | | | 557 (8) | | | | | | | | 0.098 | | | | | | | | 0.019 | | | | | 36 (9) | | | 38 (10) | | | 0.084 | | | 0.017 |
| stroke | 2,443 (36) | | | | | | 2,457 (36) | | | | | | | | 0.027 | | | | | | | | 0.004 | | | | | 100 (26) | | | 106 (28) | | | 0.032 | | | 0.035 |
| thromboembolism | 354 (5) | | | | | | 328 (5) | | | | | | | | 0.025 | | | | | | | | 0.018 | | | | | 20 (5) | | | 28 (7) | | | 0.178 | | | 0.084 |
| arterial diseases**^*^** | 1,304 (19) | | | | | | 1,270 (19) | | | | | | | | 0.025 | | | | | | | | 0.013 | | | | | 75 (20) | | | 66 (17) | | | 0.103 | | | 0.06 |
| heart failure | 2,945 (43) | | | | | | 3,027 (45) | | | | | | | | 0.085 | | | | | | | | 0.024 | | | | | 192 (51) | | | 188 (50) | | | 0.006 | | | 0.021 |
| CKD | 209 (3) | | | | | | 220 (3) | | | | | | | | 0.119 | | | | | | | | 0.008 | | | | | 15 (4) | | | 19 (5) | | | 0.259 | | | 0.043 |
| CHA2DS2-VASc |  | | | | | |  | | | | | | | |  | | | | | | | |  | | | | |  | | |  | | |  | | |  |
| 0-1, n(%) | 890 (13) | | | | | | 838 (12) | | | | | | | | 0.098 | | | | | | | | 0.024 | | | | | 52 (14) | | | 52 (14) | | | 0.004 | | | <0.001 |
| 2-3, n(%) | 2,509 (37) | | | | | | 2,479 (37) | | | | | | | | 0.011 | | | | | | | | 0.009 | | | | | 132 (35) | | | 116 (31) | | | 0.124 | | | 0.091 |
| ≥4, n(%) | 3,380 (50) | | | | | | 3,462 (51) | | | | | | | | 0.074 | | | | | | | | 0.024 | | | | | 195 (51) | | | 211 (56) | | | 0.119 | | | 0.085 |
| Medications, n(%) |  | | | | | |  | | | | | | | |  | | | | | | | |  | | | | |  | | |  | | |  | | |  |
| antiplatelet agent | 2,454 (36) | | | | | | 2,444 (36) | | | | | | | | 0.007 | | | | | | | | 0.003 | | | | | 139 (37) | | | 144 (38) | | | 0.061 | | | 0.028 |
| statin | 4,285 (63) | | | | | | 4,279 (63) | | | | | | | | 0.011 | | | | | | | | 0.002 | | | | | 193 (51) | | | 200 (53) | | | 0.01 | | | 0.037 |
| ACEI/ARB | 3,832 (57) | | | | | | 3,852 (57) | | | | | | | | 0.016 | | | | | | | | 0.006 | | | | | 198 (52) | | | 205 (54) | | | 0.045 | | | 0.037 |
| beta blocker | 3,258 (48) | | | | | | 3,325 (49) | | | | | | | | 0.052 | | | | | | | | 0.02 | | | | | 191 (50) | | | 194 (51) | | | 0.054 | | | 0.016 |
| CCB | 2,837 (42) | | | | | | 2,903 (43) | | | | | | | | 0.043 | | | | | | | | 0.02 | | | | | 153 (40) | | | 158 (42) | | | 0.007 | | | 0.027 |
| Diuretics | 612 (9) | | | | | | 656 (10) | | | | | | | | 0.020 | | | | | | | | 0.014 | | | | | 44 (12) | | | 46 (12) | | | 0.015 | | | 0.015 |
| **Characteristics** | **Stroke** | | | | | | | | | | | | | | | | | | | | | | | | | | | | | | | | | | | | |
|  | **High adherence group** | | | | | | | | | | | | | | | | | | | | | | | | | | | **Low adherence group** | | | | | | | | | |
|  | Dabigatran | | | | | | | Apixaban | | | | | | | | *d_before_* | | | | | | | | *d_after_* | | | | Dabigatran | | | Apixaban | | | *d_before_* | | | *d_after_* |
| Subjects, n | 6,779 | | | | | | | 6,779 | | | | | | | |  | | | | | | | |  | | | | 379 | | | 379 | | |  | | |  |
| Age, years | 71 ± 11 | | | | | | | 72 ± 11 | | | | | | | | 0.209 | | | | | | | | 0.065 | | | | 72 ± 13 | | | 72 ± 13 | | | 0.137 | | | 0.044 |
| Male, n(%) | 3,790 (56) | | | | | | | 3,639 (54) | | | | | | | | 0.162 | | | | | | | | 0.045 | | | | 203 (54) | | | 203 (54) | | | 0.016 | | | <0.001 |
| Medical history, n(%) |  | | | | | | |  | | | | | | | |  | | | | | | | |  | | | |  | | |  | | |  | | |  |
| hypertension | 5,404 (80) | | | | | | | 5,429 (80) | | | | | | | | 0.026 | | | | | | | | 0.009 | | | | 288 (76) | | | 292 (77) | | | 0.051 | | | 0.025 |
| diabetes | 1,723 (25) | | | | | | | 1,757 (26) | | | | | | | | 0.023 | | | | | | | | 0.011 | | | | 88 (23) | | | 84 (22) | | | 0.025 | | | 0.025 |
| dyslipidemia | 4,232 (62) | | | | | | | 4,247 (63) | | | | | | | | 0.029 | | | | | | | | 0.005 | | | | 189 (50) | | | 182 (48) | | | 0.053 | | | 0.037 |
| myocardial infarction | 516 (8) | | | | | | | 554 (8) | | | | | | | | 0.097 | | | | | | | | 0.02 | | | | 39 (10) | | | 40 (11) | | | 0.093 | | | 0.009 |
| stroke | 2,441 (36) | | | | | | | 2,467 (36) | | | | | | | | 0.027 | | | | | | | | 0.008 | | | | 111 (29) | | | 106 (28) | | | 0.039 | | | 0.029 |
| thromboembolism | 354 (5) | | | | | | | 323 (5) | | | | | | | | 0.023 | | | | | | | | 0.021 | | | | 21 (6) | | | 26 (7) | | | 0.179 | | | 0.052 |
| arterial diseases**^*^** | 1,299 (19) | | | | | | | 1,291 (19) | | | | | | | | 0.025 | | | | | | | | 0.003 | | | | 73 (19) | | | 65 (17) | | | 0.104 | | | 0.054 |
| heart failure | 2,944 (43) | | | | | | | 3,020 (45) | | | | | | | | 0.085 | | | | | | | | 0.023 | | | | 184 (49) | | | 187 (49) | | | 0.006 | | | 0.016 |
| CKD | 210 (3) | | | | | | | 211 (3) | | | | | | | | 0.118 | | | | | | | | <0.001 | | | | 15 (4) | | | 20 (5) | | | 0.268 | | | 0.054 |
| CHA2DS2-VASc |  | | | | | | |  | | | | | | | |  | | | | | | | |  | | | |  | | |  | | |  | | |  |
| 0-1, n(%) | 894 (13) | | | | | | | 821 (12) | | | | | | | | 0.097 | | | | | | | | 0.033 | | | | 51 (13) | | | 52 (14) | | | 0.003 | | | 0.008 |
| 2-3, n(%) | 2,501 (37) | | | | | | | 2,456 (36) | | | | | | | | 0.011 | | | | | | | | 0.014 | | | | 131 (35) | | | 114 (30) | | | 0.133 | | | 0.097 |
| ≥4, n(%) | 3,384 (50) | | | | | | | 3,502 (52) | | | | | | | | 0.073 | | | | | | | | 0.035 | | | | 197 (52) | | | 213 (56) | | | 0.127 | | | 0.086 |
| Medications, n(%) |  | | | | | | |  | | | | | | | |  | | | | | | | |  | | | |  | | |  | | |  | | |  |
| antiplatelet agent | 2,456 (36) | | | | | | | 2,462 (36) | | | | | | | | 0.007 | | | | | | | | 0.002 | | | | 133 (35) | | | 143 (38) | | | 0.063 | | | 0.056 |
| statin | 4,281 (63) | | | | | | | 4,309 (64) | | | | | | | | 0.011 | | | | | | | | 0.009 | | | | 201 (53) | | | 197 (52) | | | 0.012 | | | 0.021 |
| ACEI/ARB | 3,835 (57) | | | | | | | 3,869 (57) | | | | | | | | 0.017 | | | | | | | | 0.01 | | | | 207 (55) | | | 203 (54) | | | 0.043 | | | 0.021 |
| beta blocker | 3,254 (48) | | | | | | | 3,363 (50) | | | | | | | | 0.052 | | | | | | | | 0.032 | | | | 187 (49) | | | 193 (51) | | | 0.067 | | | 0.032 |
| CCB | 2,833 (42) | | | | | | | 2,834 (42) | | | | | | | | 0.043 | | | | | | | | <0.001 | | | | 159 (42) | | | 158 (42) | | | 0.008 | | | 0.005 |
| Diuretics | 611 (9) | | | | | | | 619 (9) | | | | | | | | 0.085 | | | | | | | | 0.004 | | | | 46 (12) | | | 47 (12) | | | 0.023 | | | 0.008 |
| **Characteristics** | **AMI** | | | | | | | | | | | | | | | | | | | | | | | | | | | | | | | | | | | | |
|  | **High adherence group** | | | | | | | | | | | | | | | | | | | | | | | | | | | **Low adherence group** | | | | | | | | | |
|  | Dabigatran | | | | | | | Apixaban | | | | | | | | *d_before_* | | | | | | | | *d_after_* | | | | Dabigatran | | | Apixaban | | | *d_before_* | | | *d_after_* |
| Subjects, n | 6,787 | | | | | | | 6,787 | | | | | | | |  | | | | | | | |  | | | | 367 | | | 367 | | |  | | |  |
| Age, years | 71 ± 11 | | | | | | | 72 ± 11 | | | | | | | | 0.209 | | | | | | | | 0.076 | | | | 72 ± 13 | | | 72 ± 14 | | | 0.120 | | | 0.042 |
| Male, n(%) | 3,796 (56) | | | | | | | 3,646 (54) | | | | | | | | 0.163 | | | | | | | | 0.044 | | | | 197 (54) | | | 200 (54) | | | 0.040 | | | 0.016 |
| Medical history, n(%) |  | | | | | | |  | | | | | | | |  | | | | | | | |  | | | |  | | |  | | |  | | |  |
| hypertension | 5,399 (80) | | | | | | | 5,428 (80) | | | | | | | | 0.028 | | | | | | | | 0.011 | | | | 276 (75) | | | 280 (76) | | | 0.029 | | | 0.026 |
| diabetes | 1,715 (25) | | | | | | | 1,754 (26) | | | | | | | | 0.023 | | | | | | | | 0.013 | | | | 83 (23) | | | 86 (23) | | | 0.061 | | | 0.019 |
| dyslipidemia | 4,224 (62) | | | | | | | 4,244 (63) | | | | | | | | 0.025 | | | | | | | | 0.006 | | | | 178 (49) | | | 180 (49) | | | 0.072 | | | 0.011 |
| myocardial infarction | 512 (8) | | | | | | | 534 (8) | | | | | | | | 0.100 | | | | | | | | 0.011 | | | | 35 (10) | | | 39 (11) | | | 0.05 | | | 0.035 |
| stroke | 2,414 (36) | | | | | | | 2,416 (36) | | | | | | | | 0.026 | | | | | | | | <0.001 | | | | 105 (29) | | | 105 (29) | | | 0.005 | | | <0.001 |
| thromboembolism | 354 (5) | | | | | | | 331 (5) | | | | | | | | 0.024 | | | | | | | | 0.016 | | | | 19 (5) | | | 25 (7) | | | 0.142 | | | 0.065 |
| arterial diseases**^*^** | 1,296 (19) | | | | | | | 1,303 (19) | | | | | | | | 0.025 | | | | | | | | 0.003 | | | | 71 (19) | | | 67 (18) | | | 0.098 | | | 0.028 |
| heart failure | 2,924 (43) | | | | | | | 2,988 (44) | | | | | | | | 0.054 | | | | | | | | 0.019 | | | | 184 (50) | | | 186 (51) | | | 0.008 | | | 0.011 |
| CKD | 210 (3) | | | | | | | 228 (3) | | | | | | | | 0.117 | | | | | | | | 0.013 | | | | 16 (4) | | | 23 (6) | | | 0.232 | | | 0.078 |
| CHA2DS2-VASc |  | | | | | | |  | | | | | | | |  | | | | | | | |  | | | |  | | |  | | |  | | |  |
| 0-1, n(%) | 899 (13) | | | | | | | 819 (12) | | | | | | | | 0.099 | | | | | | | | 0.037 | | | | 47 (13) | | | 51 (14) | | | 0.011 | | | 0.032 |
| 2-3, n(%) | 2,516 (37) | | | | | | | 2,504 (37) | | | | | | | | 0.012 | | | | | | | | 0.004 | | | | 122 (33) | | | 118 (32) | | | 0.104 | | | 0.023 |
| ≥4, n(%) | 3,372 (50) | | | | | | | 3,464 (51) | | | | | | | | 0.076 | | | | | | | | 0.027 | | | | 198 (54) | | | 198 (54) | | | 0.090 | | | <0.001 |
| Medications, n(%) |  | | | | | | |  | | | | | | | |  | | | | | | | |  | | | |  | | |  | | |  | | |  |
| antiplatelet agent | 2,473 (36) | | | | | | | 2,471 (36) | | | | | | | | 0.008 | | | | | | | | <0.001 | | | | 133 (36) | | | 142 (39) | | | 0.085 | | | 0.051 |
| statin | 4,278 (63) | | | | | | | 4,307 (63) | | | | | | | | 0.009 | | | | | | | | 0.009 | | | | 189 (51) | | | 195 (53) | | | 0.042 | | | 0.033 |
| ACEI/ARB | 3,828 (56) | | | | | | | 3,863 (57) | | | | | | | | 0.016 | | | | | | | | 0.010 | | | | 194 (53) | | | 191 (52) | | | 0.018 | | | 0.016 |
| beta blocker | 3,267 (48) | | | | | | | 3,304 (49) | | | | | | | | 0.049 | | | | | | | | 0.011 | | | | 182 (50) | | | 186 (51) | | | 0.087 | | | 0.022 |
| CCB | 2,836 (42) | | | | | | | 2,887 (43) | | | | | | | | 0.044 | | | | | | | | 0.015 | | | | 150 (41) | | | 147 (40) | | | 0.048 | | | 0.017 |
| Diuretics | 607 (9) | | | | | | | 635 (9) | | | | | | | | 0.083 | | | | | | | | 0.014 | | | | 46 (13) | | | 45 (12) | | | 0.010 | | | 0.008 |
| **Characteristics** | **Death** | | | | | | | | | | | | | | | | | | | | | | | | | | | | | | | | | | | | |
|  | **High adherence group** | | | | | | | | | | | | | | | | | | | | | | | | | | | **Low adherence group** | | | | | | | | | |
|  | Dabigatran | | | | | | | | Apixaban | | | | | | | | *d_before_* | | | | | | | | *d_after_* | | | Dabigatran | | | Apixaban | | | *d_before_* | | | *d_after_* |
| Subjects, n | 6,783 | | | | | | | | 6,783 | | | | | | | |  | | | | | | | |  | | | 365 | | | 365 | | |  | | |  |
| Age, years | 71 ± 11 | | | | | | | | 72 ± 11 | | | | | | | | 0.209 | | | | | | | | 0.071 | | | 72 ± 13 | | | 72 ± 14 | | | 0.122 | | | 0.025 |
| Male, n(%) | 3,796 (56) | | | | | | | | 3,629 (54) | | | | | | | | 0.164 | | | | | | | | 0.049 | | | 187 (51) | | | 198 (54) | | | 0.044 | | | 0.060 |
| Medical history, n(%) |  | | | | | | | |  | | | | | | | |  | | | | | | | |  | | |  | | |  | | |  | | |  |
| hypertension | 5,393 (80) | | | | | | | | 5,421 (80) | | | | | | | | 0.027 | | | | | | | | 0.01 | | | 280 (77) | | | 280 (77) | | | 0.033 | | | <0.001 |
| diabetes | 1,716 (25) | | | | | | | | 1,737 (26) | | | | | | | | 0.023 | | | | | | | | 0.007 | | | 79 (22) | | | 86 (24) | | | 0.063 | | | 0.045 |
| dyslipidemia | 4,228 (62) | | | | | | | | 4,234 (62) | | | | | | | | 0.025 | | | | | | | | 0.002 | | | 184 (50) | | | 177 (48) | | | 0.075 | | | 0.038 |
| myocardial infarction | 507 (7) | | | | | | | | 532 (8) | | | | | | | | 0.099 | | | | | | | | 0.013 | | | 39 (11) | | | 39 (11) | | | 0.059 | | | <0.001 |
| stroke | 2,424 (36) | | | | | | | | 2,416 (36) | | | | | | | | 0.025 | | | | | | | | 0.002 | | | 105 (29) | | | 97 (27) | | | 0.012 | | | 0.049 |
| thromboembolism | 353 (5) | | | | | | | | 333 (5) | | | | | | | | 0.022 | | | | | | | | 0.014 | | | 21 (6) | | | 26 (7) | | | 0.143 | | | 0.055 |
| arterial diseases**^*^** | 1,309 (19) | | | | | | | | 1,308 (19) | | | | | | | | 0.027 | | | | | | | | <0.001 | | | 63 (17) | | | 64 (18) | | | 0.098 | | | 0.007 |
| heart failure | 2,933 (43) | | | | | | | | 3,039 (45) | | | | | | | | 0.087 | | | | | | | | 0.032 | | | 190 (52) | | | 190 (52) | | | 0.004 | | | <0.001 |
| CKD | 211 (3) | | | | | | | | 238 (4) | | | | | | | | 0.115 | | | | | | | | 0.02 | | | 16 (4) | | | 23 (6) | | | 0.241 | | | 0.078 |
| CHA2DS2-VASc |  | | | | | | | |  | | | | | | | |  | | | | | | | |  | | |  | | |  | | |  | | |  |
| 0-1, n(%) | 899 (13) | | | | | | | | 838 (12) | | | | | | | | 0.099 | | | | | | | | 0.028 | | | 44 (12) | | | 51 (14) | | | 0.012 | | | 0.056 |
| 2-3, n(%) | 2,515 (37) | | | | | | | | 2,470 (36) | | | | | | | | 0.012 | | | | | | | | 0.014 | | | 125 (34) | | | 113 (31) | | | 0.113 | | | 0.070 |
| ≥4, n(%) | 3,369 (50) | | | | | | | | 3,475 (51) | | | | | | | | 0.076 | | | | | | | | 0.031 | | | 196 (54) | | | 201 (55) | | | 0.098 | | | 0.027 |
| Medications, n(%) |  | | | | | | | |  | | | | | | | |  | | | | | | | |  | | |  | | |  | | |  | | |  |
| antiplatelet agent | 2,466 (36) | | | | | | | | 2,466 (36) | | | | | | | | 0.008 | | | | | | | | <0.001 | | | 131 (36) | | | 140 (38) | | | 0.087 | | | 0.051 |
| statin | 4,280 (63) | | | | | | | | 4,294 (63) | | | | | | | | 0.009 | | | | | | | | 0.004 | | | 193 (53) | | | 187 (51) | | | 0.044 | | | 0.033 |
| ACEI/ARB | 3,825 (56) | | | | | | | | 3,866 (57) | | | | | | | | 0.017 | | | | | | | | 0.012 | | | 191 (52) | | | 195 (53) | | | 0.016 | | | 0.022 |
| beta blocker | 3,270 (48) | | | | | | | | 3,318 (49) | | | | | | | | 0.049 | | | | | | | | 0.014 | | | 182 (50) | | | 187 (51) | | | 0.099 | | | 0.027 |
| CCB | 2,834 (42) | | | | | | | | 2,892 (43) | | | | | | | | 0.045 | | | | | | | | 0.017 | | | 146 (40) | | | 145 (40) | | | 0.046 | | | 0.006 |
| Diuretics | 606 (9) | | | | | | | | 641 (9) | | | | | | | | 0.084 | | | | | | | | 0.017 | | | 46 (13) | | | 48 (13) | | | 0.018 | | | 0.016 |

AMI, acute myocardial infarction; NOAC, non-vitamin K antagonist oral anticoagulant; *d_before_*, standardized difference before propensity score matching; *d_after_*, standardized difference after propensity score matching; **^*^**stenosis or thrombosis of aortic and peripheral arteries; CKD, chronic kidney disease; ACEI/ARB, angiotensin-converting enzyme inhibitor or angiotensin-receptor blocker; CCB, calcium channel blocker
