# Supplementary material for: Adherence and clinical outcomes for twice-daily versus once-daily dosing of non-vitamin K antagonist oral anticoagulants in patients with atrial fibrillation: Is dosing frequency important?
Source: PLoS One. 2023 Mar 30;18(3):e0283478. doi: 10.1371/journal.pone.0283478 (PMC10062560; doi:10.1371/journal.pone.0283478)
Supplement: S6 Table — (DOCX) [file pone.0283478.s006.docx]

S6 Table. Sensitivity analyses: events and event rates in NOAC users in the overall study population and the comparative composite outcome risks of patients with low adherence compared with those with high adherence in propensity score-matched population

| **Comparative risk** | **Overall population** | | | **Propensity score-matched population**^‡^ | | |
| --- | --- | --- | --- | --- | --- | --- |
|  | **N^*^** | **Events^*^** | **Event rate per 100 PY**^†^ | **Unadjusted HR**  **(95% CI)** | **Adjusted HR**^§^  **(95% CI)** | **p value** |
| **1-year follow-up** |  |  |  |  |  |  |
| Total | 34,963/1,916 | 2,076/459 | 9.3 (7.9/58.6) | 5.86 (5.18-6.63) | 5.91 (5.22-6.70) | <0.001 |
| Once-daily regimen | 16,917/911 | 997/225 | 9.0 (7.6/55.1) | 4.99 (4.21-5.92) | 5.19 (4.35-6.18) | <0.001 |
| Twice-daily regimen | 18,046/1,005 | 1,079/234 | 9.7 (8.2/62.4) | 6.35 (5.34-7.55) | 6.56 (5.50-7.83) | <0.001 |
| Apixaban | 10,536/501 | 680/145 | 9.9 (8.4/77.6) | 6.53 (5.24-8.15) | 7.02 (5.59-8.80) | <0.001 |
| Dabigatran | 7,510/504 | 399/89 | 9.3 (7.9/47.4) | 4.58 (3.50-5.99) | 4.71 (3.58-6.19) | <0.001 |
| **2-year follow-up** |  |  |  |  |  |  |
| Total | 33,009/1,719 | 2,984/479 | 7.8 (6.9/62.7) | 6.09 (5.42-6.84) | 6.40 (5.68-7.22) | <0.001 |
| Once-daily regimen | 15,964/791 | 1,457/239 | 7.5 (6.6/60.1) | 5.75 (4.87-6.78) | 5.92 (4.99-7.04) | <0.001 |
| Twice-daily regimen | 17,045/928 | 1,527/240 | 8.2 (7.2/65.6) | 6.76 (5.72-8.00) | 7.11 (5.98-8.44) | <0.001 |
| Apixaban | 9,952/452 | 984/149 | 8.3 (7.3/86.1) | 6.88 (5.56-8.52) | 7.51 (6.03-9.36) | <0.001 |
| Dabigatran | 7,093/476 | 543/91 | 7.9 (6.9/47.2) | 4.79 (3.69-6.23) | 5.01 (3.83-6.56) | <0.001 |
| **3-year follow-up** |  |  |  |  |  |  |
| Total | 32288/1509 | 3551/417 | 7.2 (6.4/62.9) | 5.23 (4.62-5.91) | 5.40 (4.76-6.12) | <0.001 |
| Once-daily regimen | 15621/693 | 1740/206 | 6.9 (6.1/57.4) | 4.76 (4.01-5.65) | 4.97 (4.15-5.94) | <0.001 |
| Twice-daily regimen | 16667/816 | 1811/211 | 7.6 (6.8/69.5) | 5.78 (4.85-6.89) | 5.98 (5.00-7.16) | <0.001 |
| Apixaban | 9709/385 | 1188/124 | 7.7 (6.9/89.1) | 5.71 (4.56-7.16) | 6.21 (4.91-7.85) | <0.001 |
| Dabigatran | 6958/431 | 623/87 | 7.4 (6.5/51.6) | 4.55 (3.49-5.93) | 4.65 (3.54-6.10) | <0.001 |
| **30-days interval gap** |  |  |  |  |  |  |
| Total | 27,662/820 | 3,290/194 | 7.5 (7.2/107.1) | 7.57 (6.27-9.13) | 7.40 (6.09-9.00) | <0.001 |
| Once-daily regimen | 13,363/356 | 1,594/93 | 7.1 (6.7/98.8) | 6.54 (5.03-8.51) | 5.952 (4.521-7.834) | <0.001 |
| Twice-daily regimen | 14,299/464 | 1,696/101 | 8.0 (7.6/116.2) | 8.98 (6.85-9.85) | 9.42 (7.11-12.48) | <0.001 |
| Apixaban | 8,273/230 | 1,105/67 | 8.1 (7.7/163.0) | 10.54 (7.41-14.99) | 10.87 (7.50-15.76) | <0.001 |
| Dabigatran | 6,026/234 | 591/34 | 7.9 (7.5/74.2) | 4.82 (3.16-7.35) | 4.69 (3.03-7.27) | <0.001 |
| **180-days interval gap** |  |  |  |  |  |  |
| Total | 33,843/3,970 | 3,922/1,069 | 7.5 (6.2/30.2) | 3.85 (3.57-4.15) | 3.81 (3.53-4.11) | <0.001 |
| Once-daily regimen | 1,6394/1,882 | 1,920/545 | 7.3 (6.0/29.6) | 3.71 (3.34-4.12) | 3.67 (3.30-4.08) | <0.001 |
| Twice-daily regimen | 17,449/2,088 | 2,002/524 | 7.7 (6.5/30.9) | 3.69 (3.32-4.11) | 3.74 (3.36-4.16) | <0.001 |
| Apixaban | 1,0231/1,085 | 1,315/327 | 7.9 (6.6/65.5) | 4.19 (3.66-4.80) | 4.28 (3.73-4.92) | <0.001 |
| Dabigatran | 7,218/1,003 | 687/197 | 7.5 (6.2/25.4) | 3.41 (2.87-4.05) | 3.35 (2.82-3.99) | <0.001 |

NOAC, non-vitamin K antagonist oral anticoagulant; **^*^**crude number and events of patients with high adherence/low adherence; ^†^incidence rate of total patients (high adherence users/low adherence users); ^‡^matching covariates, including demographics, CHA2DS2-VASc score, and medical information data between high and low adherence users; ^§^hazard ratio (HR) after adjusting the aforementioned covariates using Cox proportional hazard models; PY, person-years; CI, confidence interval
