# Supplementary material for: Adherence and clinical outcomes for twice-daily versus once-daily dosing of non-vitamin K antagonist oral anticoagulants in patients with atrial fibrillation: Is dosing frequency important?
Source: PLoS One. 2023 Mar 30;18(3):e0283478. doi: 10.1371/journal.pone.0283478 (PMC10062560; doi:10.1371/journal.pone.0283478)
Supplement: Attachment — Submitted filename: respnse to reviewer.docx [file pone.0283478.s008.docx]

PONE-D-22-19616

Adherence and Clinical Outcomes for Twice-Daily Versus Once-Daily Dosing of Direct Oral Anticoagulants in Patients with Atrial Fibrillation: Is Dosing Frequency Important?

PLOS ONE

To the editor,

We appreciate your consideration for publication in the PLOS ONE. We sincerely revised the manuscript as Journal requirements and Reviewers' comments. In addition, the word “DOAC” was changed to “NOAC” according to the recent guidelines of atrial fibrillation (Eur Heart J 2020;42:373-498). The revised manuscript was re-edited by professional editors at Editage, a division of Cactus Communications.

**Journal Requirements:**

1. Please ensure that your manuscript meets PLOS ONE's style requirements, including those for file naming. The PLOS ONE style templates can be found at <https://journals.plos.org/plosone/s/file?id=wjVg/PLOSOne_formatting_sample_main_body.pdf> and <https://journals.plos.org/plosone/s/file?id=ba62/PLOSOne_formatting_sample_title_authors_affiliations.pdf>

Answer) The manuscript complied with the style requirements of PLOS ONE.

2. Please provide additional details regarding participant consent. In the ethics statement in the Methods and online submission information, please ensure that you have specified what type you obtained (for instance, written or verbal, and if verbal, how it was documented and witnessed). If your study included minors, state whether you obtained consent from parents or guardians. If the need for consent was waived by the ethics committee, please include this information.

Answer) Because Korean claim data are anonymized and de-identified, patients’ informed consent was waved. We add the fact in the manuscript.

(in manuscript) The hospital Ethics Committee approved this study and waived the requirement for informed consent from the patients, because claim data were provided in anonymized and de-identified form (KHNMC 2020-06-016).

3. In your Data Availability statement, you have not specified where the minimal data set underlying the results described in your manuscript can be found. PLOS defines a study's minimal data set as the underlying data used to reach the conclusions drawn in the manuscript and any additional data required to replicate the reported study findings in their entirety. All PLOS journals require that the minimal data set be made fully available. For more information about our data policy, please see <http://journals.plos.org/plosone/s/data-availability.>

"Upon re-submitting your revised manuscript, please upload your study’s minimal underlying data set as either Supporting Information files or to a stable, public repository and include the relevant URLs, DOIs, or accession numbers within your revised cover letter. For a list of acceptable repositories, please see <http://journals.plos.org/plosone/s/data-availability#loc-recommended-repositories.> Any potentially identifying patient information must be fully anonymized.

Important: If there are ethical or legal restrictions to sharing your data publicly, please explain these restrictions in detail. Please see our guidelines for more information on what we consider unacceptable restrictions to publicly sharing data: <http://journals.plos.org/plosone/s/data-availability#loc-unacceptable-data-access-restrictions.> Note that it is not acceptable for the authors to be the sole named individuals responsible for ensuring data access.

We will update your Data Availability statement to reflect the information you provide in your cover letter. If there are restrictions on publicly sharing data—e.g. participant privacy or use of data from a third party—those must be specified.

Answer) Data cannot be shared publicly because of the provisions of the National Health Insurance Service (NHIS). Korean legal restrictions prohibit authors from making the data publicly available, and the authority implemented the restrictions is NHIS (National Health Insurance Service), one of the government agency of Republic of Korea. NHIS provides limited portion of anonymized data to the researchers for the purpose of the public interest. However, they exclusively provide data to whom made direct contact of the NHIS and agreed to policies of NHIS. Redistribution of the data is not permitted for the researchers. We add the fact in the manuscript.

(in manuscript) Data supporting this study are publicly available at the NHIS webpage (<http://nhiss.nhis.or.kr>).

**Reviewers' comments:**
Reviewer #1: I read the manuscript with interest, also for the practical impact in our daily activity. Adherence to anticoagulation therapy (i.e. DOACs) is critical in patients with non-valvular AF and, the authors have rightly pointed out the need to promote a higher adherence among patients. Overall, the data of this study constitute an important information about how to properly manage anti thrombotic medication in patients with non-valvular AF

Answer) Thank you for your good estimation.

Reviewer #2: The paper of L SUK Sohn discusses the relevant issue of the impact of adherence to DOAC treatment on different drug regimens. They found no impact of once a day vs. twice a day administration in terms of adherence, while in both cases the clinical outcomes were worst in low adherence cohort.
The topic is relevant ad results interesting. However, there are some methodological concerns.

1. We have no data on dose prescription according to smpc. I particular the number of subjects treated with low doses seems quite high in general and this is particularly relevant for apixaban considering the small amount of patients receiving low doses in the registration trial.

Answer) Based on your comments, we added the definition on usage and dosage of standard and low dose NOACs. The percentage of low-dose NOACs was high, ranging from 40 to 60%, which was higher in low-dose dabigatran users than apixaban users (Table 1). Clinical outcomes in the low adherence group were consistently worse in low- and standard- dose NOAC users (Supple Fig 1).

(in manuscript) The standard-dose NOACs were defined as 5 mg apixaban and 150 mg dabigatran twice daily and 60 mg edoxaban and 20 mg rivaroxaban once daily. The low-dose NOACs were defined as 2.5 mg apixaban and 110 mg dabigatran twice daily and 30 mg edoxaban and 15 mg rivaroxaban once daily. Patients who were prescribed very low doses of NOACs (e.g., 15 mg edoxaban or 10 mg rivaroxaban) or once-daily dosing of apixaban or dabigatran were excluded.

2. Authors show only CKD prevalence which is really low for this type of population whereas it has a big impact on clinical outcomes and adherence due to both ischemic and hemorragic events.

Answer) Patients with end-stage renal disease were excluded in this study (Fig 1). Furthermore, NOAC use is restricted in CKD patients with creatinine clearance < 15-30 mg/min. Thus, CKD rate was low in the study enrollment population (3-6% in Table 1). It is clear that CKD is an important risk factor for clinical outcomes. Thus, CKD was used as a covariate on propensity score and multivariate Cox proportional hazard models for clinical outcome assessment (S2 – 5 Tables).

3. It is not clear the use of propensity score matching because it seems to be used to compare patients with low vs. high adherence especially in table 2 but looking at the methods section and considering the comparison between different agents in the subgroup of patients with high adherence it is not clear if this comparison was made on propensity matched cohorts. If this was done, clinical characteristics of the matched cohorts should be reported otherwise the comparison between cohorts non directly matched should be omitted.

Answer) S5 table shows the standardized differences before and after propensity score matching of clinical characteristics between each NOAC in figure 5 (comparative clinical outcomes of each NOAC in the high and low adherence groups), in which matched clinical data including demographics are skipped due to their high volume. However, as per your recommendation, we revised them in the S5 table. Similarly, S2 – S4 tables show standardized differences and/or clinical data before and after propensity score matching to compare clinical outcomes between the low and high adherence groups (this is, clinical data of propensity score matched population supporting Table 2 results). In S4 table, we added clinical data after propensity score matching between the two groups.
